# Mutant p53^L194F^ Harboring Luminal-A Breast Cancer Cells Are Refractory to Apoptosis and Cell Cycle Arrest in Response to Mortaparib^Plus^, a Multimodal Small Molecule Inhibitor

**DOI:** 10.3390/cancers13123043

**Published:** 2021-06-18

**Authors:** Ahmed Elwakeel, Anissa Nofita Sari, Jaspreet Kaur Dhanjal, Hazna Noor Meidinna, Durai Sundar, Sunil C. Kaul, Renu Wadhwa

**Affiliations:** 1 AIST-INDIA DAILAB, National Institute of Advanced Industrial Science & Technology (AIST), Tsukuba, Ibaraki 305-8565, Japan; elwakeela@uni.coventry.ac.uk (A.E.); sari-anissa@aist.go.jp (A.N.S.); jaspreet@iiitd.ac.in (J.K.D.); hazna.meidinna@aist.go.jp (H.N.M.); 2 School of Integrative & Global Majors (SIGMA), University of Tsukuba, Tsukuba 305-8577, Japan; 3 DAILAB, Department of Biochemical Engineering & Biotechnology, Indian Institute of Technology (IIT) Delhi, Hauz Khas, New Delhi 110-016, India; sundar@dbeb.iitd.ac.in

**Keywords:** Mortaparib^Plus^, luminal-A breast cancer cells, mortalin–p53 interaction, PARP1 hyperactivation, DNA damage, ATP, tumor suppression

## Abstract

**Simple Summary:**

Tumor suppressor protein p53 is a master regulator that inhibits the process of oncogenesis by induction of cell senescence/cell cycle arrest/apoptosis during normal and stressed states of cells. It is functionally inactivated in the majority of cancers. Mortalin, a member of the Hsp70 family of proteins, enriched in cancer cells, is known to cause cytoplasmic sequestration and inactivation of the p53’s transcriptional activation function. Inhibition of mortalin–p53 interaction and reactivation of p53 functions by natural and synthetic drugs has emerged as a possible cancer therapeutic strategy. We recently reported a novel multimodal small molecule, named Mortaparib^Plus^, that inhibited mortalin–p53 interaction and caused reactivation of p53 function in colorectal cancer cells. Here, we report its effect on breast cancer cells with wildtype (MCF-7) or mutant (T47D) p53 status.

**Abstract:**

We previously performed a drug screening to identify a potential inhibitor of mortalin–p53 interaction. In four rounds of screenings based on the shift in mortalin immunostaining pattern from perinuclear to pan-cytoplasmic and nuclear enrichment of p53, we had identified Mortaparib^Plus^ (4-[(1E)-2-(2-phenylindol-3-yl)-1-azavinyl]-1,2,4-triazole) as a novel synthetic small molecule. In order to validate its activity and mechanism of action, we recruited Luminal-A breast cancer cells, MCF-7 (p53^wild type^) and T47D (p53^L194F^) and performed extensive biochemical and immunocytochemical analyses. Molecular analyses revealed that Mortaparib^Plus^ is capable of abrogating mortalin–p53 interaction in both MCF-7 and T47D cells. Intriguingly, upregulation of transcriptional activation function of p53 (as marked by upregulation of the p53 effector gene—*p21^WAF1^*—responsible for cell cycle arrest and apoptosis) was recorded only in Mortaparib^Plus^-treated MCF-7 cells. On the other hand, Mortaparib^Plus^-treated T47D cells exhibited hyperactivation of PARP1 (accumulation of PAR polymer and decrease in ATP levels) as a possible non-p53 tumor suppression program. However, these cells did not show full signs of either apoptosis or PAR-Thanatos. Molecular analyses attributed such a response to the inability of Mortaparib^Plus^ to disrupt the AIF–mortalin complexes; hence, AIF did not translocate to the nucleus to induce chromatinolysis and DNA degradation. These data suggested that the cancer cells possessing enriched levels of such complexes may not respond to Mortaparib^Plus^. Taken together, we report the multimodal anticancer potential of Mortaparib^Plus^ that warrants further attention in laboratory and clinical studies.

## 1. Introduction

Luminal-A breast cancer represents 40–50% of all breast cancer patients based on the immunohistochemistry-based classification [1]. It is an estrogen-receptor-positive (ER+) subtype of the breast adenocarcinoma. Initially, it was classified as “luminal” subtype because it expresses the genes transcribed by the estrogen receptor (ER) nuclear transcription factor. These genes are typically expressed in the “luminal epithelium” that lines the mammary ducts. Luminal-A breast cancer patients have a good prognosis with a lower relapse rate than in other subtypes [2]. The “gold standard” treatment for this subtype of breast carcinoma is endocrine therapy (or anti-estrogen therapy), which is designed to block the estrogen receptor signaling pathway through Selective Estrogen Receptor Modulators (SERM) (Tamoxifen and Raloxifene), Selective Estrogen Receptor Down-regulators (SERD) (Fulvestrant) and Aromatase Inhibitors (Anastrozole) [1]. Despite the high sensitivity or responsiveness of Luminal-A breast cancers to endocrine therapies, it has an intrinsic or acquired resistance [1,3]. Anti-estrogen resistance has a molecular basis that enables cancer cells to proliferate regardless of the availability of estrogen as a ligand, for instance, the mis-sense mutations in Estrogen Receptor (Tyr537Asn, Tyr537Ser, Asp538Gly, Leu536Gln and Glu380Gln) that lead to constitutive transactivation activity in the absence of the ligand. Furthermore, amplification of the estrogen receptor gene sequence (ESR1) was previously reported to be correlated to the acquired resistance to anti-estrogen therapies [4,5]. Additionally, from a recent comprehensive profiling of the mutational statuses and expression level of DNA Damage Repair (DDR) genes in ER^+^ breast tumors, DDR defects are considered as a novel driver of endocrine therapy resistance and open new doors for alternative therapeutic possibilities [6]. Hence, novel therapeutic strategies are still required to combat the acquired or innate resistance for the Luminal-A breast cancers.

Mortalin/GRP75/HSPA9/mthsp70 is a member of the heat shock protein 70 family of chaperones. It has been established to execute essential mitochondrial and extra-mitochondrial functions including protein folding, chaperoning, intracellular trafficking, mitochondrial biogenesis, centrosome duplication and others [7,8,9,10,11,12]. Elevated mortalin levels in multiple cancerous tissues and tumor-derived cell lines emphasized its key role in oncogenesis, the epithelial-to-mesenchymal transition (EMT) and cancer cell stemness [13,14,15,16]. One of its oncogenic roles involves binding to the tumor suppressor protein p53 and causing its retention in the cell cytoplasm [17,18,19]. Mortalin–p53 interaction was found to be cancer-specific and hence serve as a selective target for an alternative cancer therapy [20]. Previously, some natural compounds (Withaferin A, Embelin and CAPE) have been defined to possess potential to block mortalin–p53 interaction [21,22,23]. These compounds were shown to relocate and reactivate the tumor suppressor function of p53 yielding growth arrest or apoptosis in cancer cells [23,24,25].

Poly-ADP-Ribose Polymerase 1 (PARP1) is a 116-kDa protein that localizes in the nucleus in response to DNA damage. Upon mild or moderate DNA damage, it is recruited to damage sites through its zinc-finger DNA-binding capability. Then, through its catalytic capability, it catalyzes the addition of linear and/or branched chains of ADP-Ribose (Poly ADP-Ribose (PAR)) to itself and to PAR-binding motifs on acceptor histone and non-histone proteins in processes called “auto-PARylation” and “hetero-PARylation”, respectively. Auto-PARylated PARP1 recruits other proteins to bind non-covalently to PAR through their PAR-binding modules at the sites of DNA-damage, initiating an intricate repair machinery. Eventually, PARP1 affinity for DNA is reduced followed by its release from the damage sites to be accessed by other repair proteins. However, upon profound DNA damage, PARP1 is hyperactivated to produce long-chained, branched PAR polymers leading to a form of cell death called PARP1-mediated cell death or “PAR-Thanatos” [26]. Although PARP1 was originally discovered as a key player of the Base Excision Repair (BER) of the Single-Stranded Break (SSB) repair pathway, now, it is known to have broader roles in the major DNA repair pathways [27,28]. PARP1 inhibitors compete with the Nicotinamide Adenine Dinucleotide (NAD^+^) substrate at the catalytic site of PARP1 preventing both the hetero-PARylation of target proteins and the PARP1 auto-PARylation processes. Inhibition of PARylation of DNA damage repair acceptor proteins results in the accumulation of DNA Double-Strand Breaks (DSB) leading to cancer cell death. Inhibition of PARP1 auto-PARylation by PARP inhibitors (PARPi), without interfering with its DNA binding capability, results in its “trapping” at damage sites. PARP1 trapping causes excessive DNA Double-Strand Breaks (DSB) during the S-phase of the cell cycle by the collapse of stalled replication forks supporting cancer cell death [28]. PARP1 inhibitors are used for the treatment of cancers with specific genetic anomalies that make them susceptible to DNA damage in the so-called “chemical synthetic lethality”. Hence, PARP1 inhibitors have achieved major clinical successes in the treatment of cancers with defects in the Homologous Recombination DNA repair pathway associated with BRCA1 and BRCA2 mutations or when combined with traditional DNA damaging agents such as chemotherapy and/or radiotherapy [29].

In luminal-A breast cancer, the role of ER-α is essential in oncogenesis (cell survival, proliferation and tumor growth). Such a role is significant enough to tip the balance of “ER-α-mediated proliferation/p53-mediated suppression” towards an uncontrolled tumor growth. Hence, activation of p53 signaling through the liberation from mortalin complex could be a successful strategy to overcome the Luminal-A breast cancers with the de novo and/or the acquired resistance to endocrine therapy. Accordingly, using MCF-7 Luminal-A breast cancer cells, a screening system was established in search of potential p53-mortalin interaction abrogators [30]. After several screening cycles, 4-[(1E)-2-(2-phenylindol-3-yl)-1-azavinyl]-1,2,4-triazole (Mortaparib^Plus^) was identified and validated to be effective for colorectal cancer cells [31]. In the present study, we report that whereas Mortaparib^Plus^ caused reactivation of p53 function in wild type p53 possessing luminal breast cancer cells, p53^L194F^ mutant possessing cells were refractory. However, they exhibited Mortaparib^Plus^-induced hyperactivation of PARP1, yet another molecular mechanism of action of Mortaparib^Plus^ that is relevant to its anticancer activity in p53-unresponsive cancer cells.

## 2. Results

### 2.1. Mortaparib^Plus^ Blocked Mortalin–p53 Interaction in Luminal-A Breast Cancer Cells

**Figure 1:**
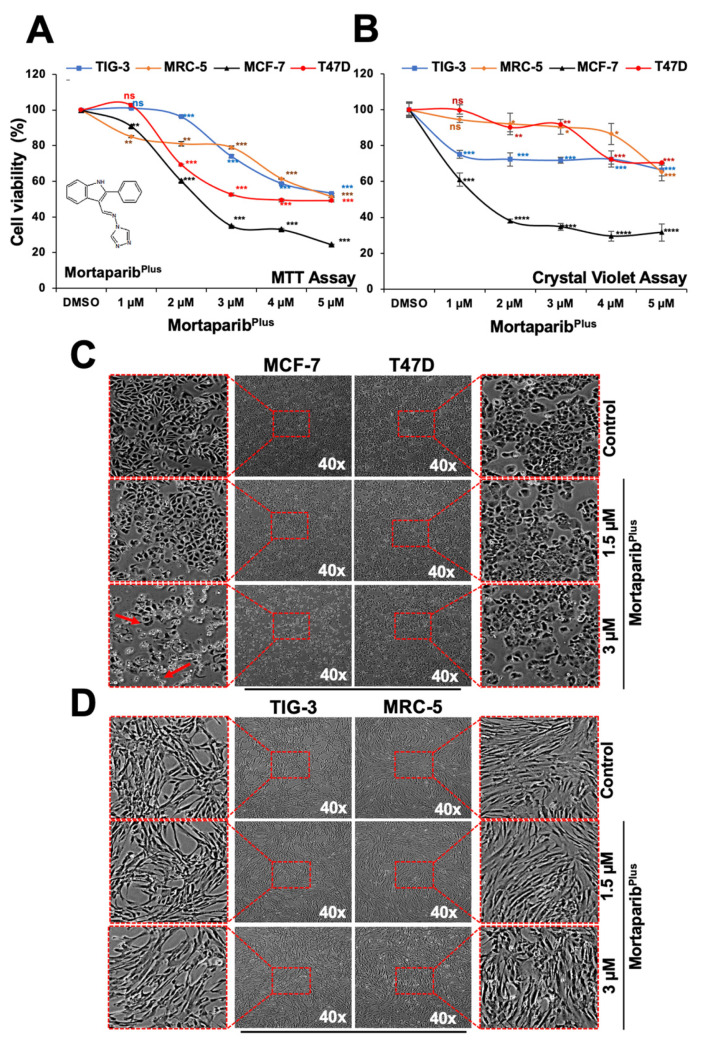
Mortaparib^Plus^ is toxic to MCF-7, but not T47D, Luminal-A breast cancer cells. (**A**) MTT-cell viability assays for control and 48 h-Mortaparib^Plus^-treated cells showed a dose-dependent cytotoxicity in MCF-7 and T47D with a milder effect on the non-tumorigenic lung fibroblasts (TIG-3 and MRC-5). Chemical structure of Mortaparib^Plus^ is shown. (**B**) CV-cell viability assay for control and 48 h-Mortaparib^Plus^-treated cells showed dose-dependent cytotoxicity in MCF-7 cells; T47D showed poor response and were similar to MRC-5 and TIG-3. (**C**) Phase-contrast micrographs of control and 48 h-Mortaparib^Plus^-treated MCF-7 cells showed a stressed phenotype (condensation and blebbing morphologies); T47D, MRC-5 and TIG-3 cells did not show such stress/cytotoxic morphology (**C**,**D**). The quantified cell viability data represents mean ± SD obtained from independent biological replicates; *p*-values were calculated using unpaired Student’s *t*-test. * ≤0.05, ** ≤0.01, *** ≤0.001 and **** ≤0.0001 represent significant, very significant, highly significant and extremely significant, respectively.

Previously, using the wild-type p53 possessing MCF-7 breast carcinoma and U2OS osteosarcoma cells, a library of 12,000 molecules was screened to identify potential mortalin–p53 interaction disruptors based on the shift in mortalin staining pattern from perinuclear to pan-cytoplasmic and the stabilization, accumulation and nuclear enrichment of p53. In four rounds of screenings, Mortaparib [30] and Mortaparib^Plus^ [31] (Figure 1A) were identified. In the colorectal cancer (CRC) in vitro models, Mortaparib^Plus^ was validated as an abrogator of the mortalin–p53 interaction irrespective of the p53 status (wild type/mutant). In the present study, in order to confirm such a mechanism of action and valorize its potentiality in other cancer disease models, we recruited the Luminal-A breast cancer cell lines possessing either the wild-type p53^WT^ (MCF-7) or mutant-p53^L194F^ (T47D) [32]. Such recruitment was done based on the similarity between CRC and Luminal-A breast cancer in terms of the cellular and mutational characteristics [33]. First, we determined the toxic and sub-toxic doses of Mortaparib^Plus^ by MTT viability assays on both the cancer cell lines after 48 h of treatment with Mortaparib^Plus^. TIG-3 and MRC-5 lung fibroblasts were used as non-cancerous cell models (that do not possess mortalin–p53 interaction). As shown in Figure 1A, within the range of 1*–*5 μM, Mortaparib^Plus^ caused dose-dependent decrease in cell viability in both breast cancer (MCF-7 and T47D) cell lines in MTT assays. Of note, at 2*–*4 μM dose, cancer cells showed higher cytotoxicity as compared to the normal cells. In order to further confirm these findings, we also performed non-REDOX dependent cytotoxicity assays, namely Crystal Violet (CV) (Figure 1B) and Lactate Dehydrogenase (LDH) assays (Supplementary Figure S1A). These assays confirmed dose-dependent cytotoxicity of Mortaparib^Plus^ and higher sensitivity of MCF-7 cells (p53^WT^) as compared to T47D cells (p53^L194F^). Of note, low doses (1–3 μM) of Mortaparib^Plus^ were relatively non-toxic to the normal lung fibroblasts (TIG-3 and MRC-5 cells) both at the 24 h [31] and 48 h treatment times (Figure 1A) in MTT assays. However, non-REDOX (CV and LDH) assays revealed that the response of T47D cells was similar to normal lung fibroblasts, especially at low doses (1*–*3 μM). Time course CV assay in control and 3 μM Mortaparib^Plus^-treated T47D cells confirmed that these cells did not respond to the drug until 48 h followed by a slow decrease in viability (Supplementary Figure S1B). In order to get insights into the molecular mechanism of action of Mortaparib^Plus^, we selected 1.5 μM and 3 μM as subtoxic and toxic doses, respectively. We next examined the control and treated cells under the microscope. As shown in Figure 1C,D, Mortaparib^Plus^-treated MCF-7 cells (p53^WT^) showed a dramatic stress phenotype (condensation and blebbing). On the other hand, similar treatments to T47D (p53^L194F^), MRC-5 and TIG-3 did not cause such remarkable phenotypic changes suggesting that Mortaparib^Plus^ caused strong growth arrest/apoptosis in MCF-7, but not in T47D, MRC-5 and TIG-3 cells.

**Figure 2:**
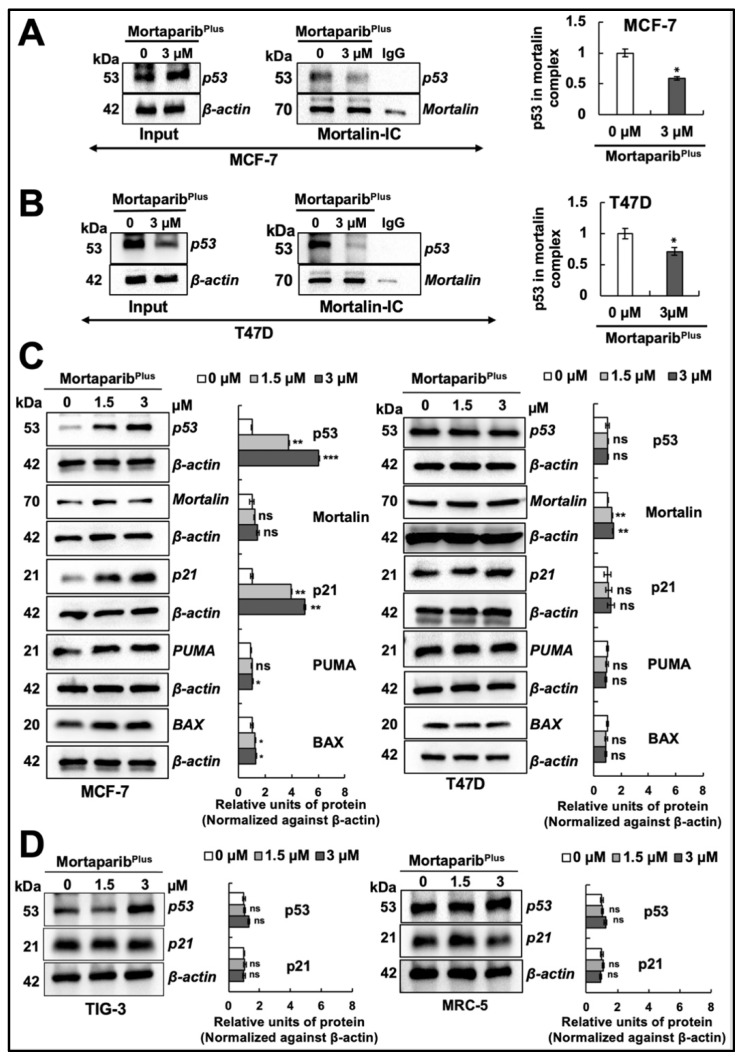
Mortaparib^Plus^ abrogated the interaction of mortalin with p53 in vitro causing the reactivation of the wild-type p53 signaling. Co-immunoprecipitation (Co-IP) analyses using a rabbit monoclonal anti-mortalin antibody (**A**,**B**) showed a decrease in p53 fractions (wild type and mutant) in mortalin complexes immunoprecipitated from Mortaparib^Plus^-treated cells. Full uncropped blots for the Co-IP experiments are shown in Supplementary Figures S5 and S6. Control and 48 h-Mortaparib^Plus^-treated cells were analyzed for the expression levels of p53, mortalin and several p53 downstream target genes by Western blotting (**C**). An accumulation in p53 levels with the overexpression of its downstream target genes (p21^WAF1^, PUMA and BAX) was observed in 48 h-Mortaparib^Plus^-treated MCF-7, but not T47D, cells as compared to the untreated control. Control and Mortaparib^Plus^-treated normal lung fibroblasts were analyzed for the expression levels of p53 and p21 by Western blotting (**D**). TIG-3 and MRC-5 normal lung fibroblasts also did not show a significant increase in p53 or its downstream effector p21^WAF1^. Full uncropped blots for the Western blotting experiments are shown in Supplementary Figures S7–S14. The quantified data represent mean ± SD obtained from independent biological replicates; *p*-values were calculated using unpaired Student’s *t*-test. ns > 0.05, * ≤0.05, ** ≤0.01 and *** ≤0.001 represent non-sgnificant, significant, very significant and highly significant, respectively.

Of note, structural homology of Mortaparib^Plus^ to the drugs currently being used for Luminal-A breast cancer patients did not show any similarity either with chemotherapeutic drugs (Supplementary Figure S2A) or anti-estrogen therapeutic drugs (Supplementary Figure S2B). Based on these data, we predicted that Mortaparib^Plus^-mediated cytotoxicity to breast cancer cells may involve a unique molecular mechanism of action. In accordance with the previously conducted computational analyses that predicted Mortaparib^Plus^ binding to mortalin, but not p53 [31], co-immunoprecipitation analyses were performed to immunoprecipitate equal amounts of mortalin complexes from the control and Mortaparib^Plus^-treated breast cancer cell lysates. As shown in Figure 2A,B, using the pantropic anti-p53 antibody (DO-1) that recognizes an epitope residue between amino acids 11 and 25 of the p53 protein (both wild-type and mutant isoforms), mortalin complexes from control and treated cells showed a decrease in p53 in the latter regardless of its status (p53^WT^ in MCF-7 cells and p53^L194F^ mutant in T47D cells).

### 2.2. Mortaparib^Plus^ Restored the Transcriptional Activation Function of the Wild-Type p53 in MCF-7 Cells Yielding Apoptosis and Cell Cycle Arrest

**Figure 3:**
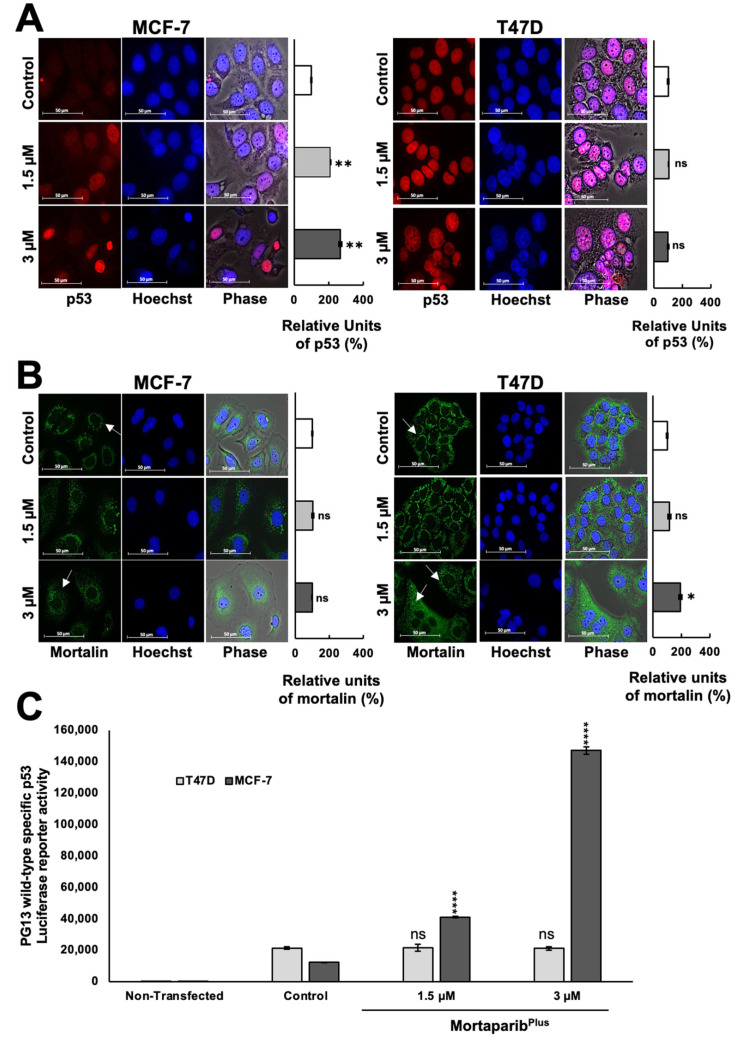
Mortaparib^Plus^ caused nuclear translocation of p53. The sub-cellular localization of p53 and mortalin in the control and 48 h-Mortaparib^Plus^-treated cells was examined by immunocytochemistry (**A**,**B**). Mortaparib^Plus^-treated MCF-7 cells showed p53 enrichment in the nuclei as compared to the nuclei of untreated control. T47D cells showed the mutant p53^L194F^ in the nuclei of both the 48 h-Mortaparib^Plus^-treated and untreated ones. The shift of the mortalin staining pattern from perinuclear in control to pan-cytoplasmic in Mortaparib^Plus^-treated MCF-7 and T47D cells was also observed (shown by white arrows). The transcriptional activation function of p53 was examined by the p53 wild-type specific luciferase reporter (PG13-Luc) assay (**C**). The wild-type p53-dependent luciferase reporter activity showed a dramatic increase in Mortaparib^Plus^-treated MCF-7, but not T47D, cells. Scale bar: 50 μm. The quantified data represent mean ± SD obtained from independent biological replicates; *p*-values were calculated using unpaired Student’s *t*-test. ns > 0.05, * ≤0.05, ** ≤0.01, and **** ≤0.0001 represent significant, non-significant, very significant, and extremely significant, respectively.

Several experiments on expression analyses by Western blotting showed the stabilization and accumulation of wild-type p53 protein in Mortaparib^Plus^-treated MCF-7 as compared to the untreated control (Figure 2C). On the other hand, the mutant p53 protein was intrinsically accumulated in the control untreated T47D cells, as previously reported [34], and there were no significant changes in its accumulation level in response to Mortaparib^Plus^ treatment (Figure 2C). Interestingly, the p53 downstream effector gene *p21^WAF1^* (the major regulator of p53-mediated cell cycle arrest) was remarkably upregulated only in Mortaparib^Plus^-treated MCF-7 cells. Mortaparib^Plus^-treated T47D cells did not show a change in p21^WAF1^ (Figure 2C). On the other hand, the level of proteins directly involved in apoptosis (BAX and PUMA) showed a minor increase in Mortaparib^Plus^-treated MCF-7, but not T47D, cells (Figure 2C,D). We also performed a similar expression assay on normal lung fibroblasts. As shown in Figure 2D (representative Western blot and quantitation obtained from three independent sets of experiments), Mortaparib^Plus^ did not cause any remarkable difference in the expression level of p53 or its downstream effector p21^WAF1^ in either TIG-3 or MRC-5 cells. We next examined the sub-cellular localization of the accumulated p53 protein and the mortalin staining pattern in control and Mortaparib^Plus^-treated cells by immunocytochemistry. As shown in Figure 3A, whereas p53 staining was negligible in the control untreated MCF-7 (p53^WT^) cells, Mortaparib^Plus^-treated cells showed clear p53 enrichment in the nuclei. On the other hand, T47D cells showed almost equal intensity of mutant p53^L194F^ in the nuclei of both the untreated and treated cells. Of note, as shown in Figure 3B, the shift in mortalin staining pattern from perinuclear in control to pan-cytoplasmic in treated cells was confirmed in both MCF-7 and T47D cells. Furthermore, we performed p53 wild-type specific luciferase reporter (PG13-Luc) assay to determine the transcriptional activation function of p53. As shown in Figure 3C, the wild-type p53-dependent luciferase reporter activity showed a dramatic increase in Mortaparib^Plus^ (both 1.5 μM and 3 μM)-treated MCF-7, but not T47D, cells. Taken together, these data suggested that Mortaparib^Plus^ abrogated mortalin–p53 interactions in MCF-7 as well as T47D cells (Figure 2A,B); however, only MCF-7 (wild type p53) showed stimulation of the transcriptional activation function of p53.

**Figure 4:**
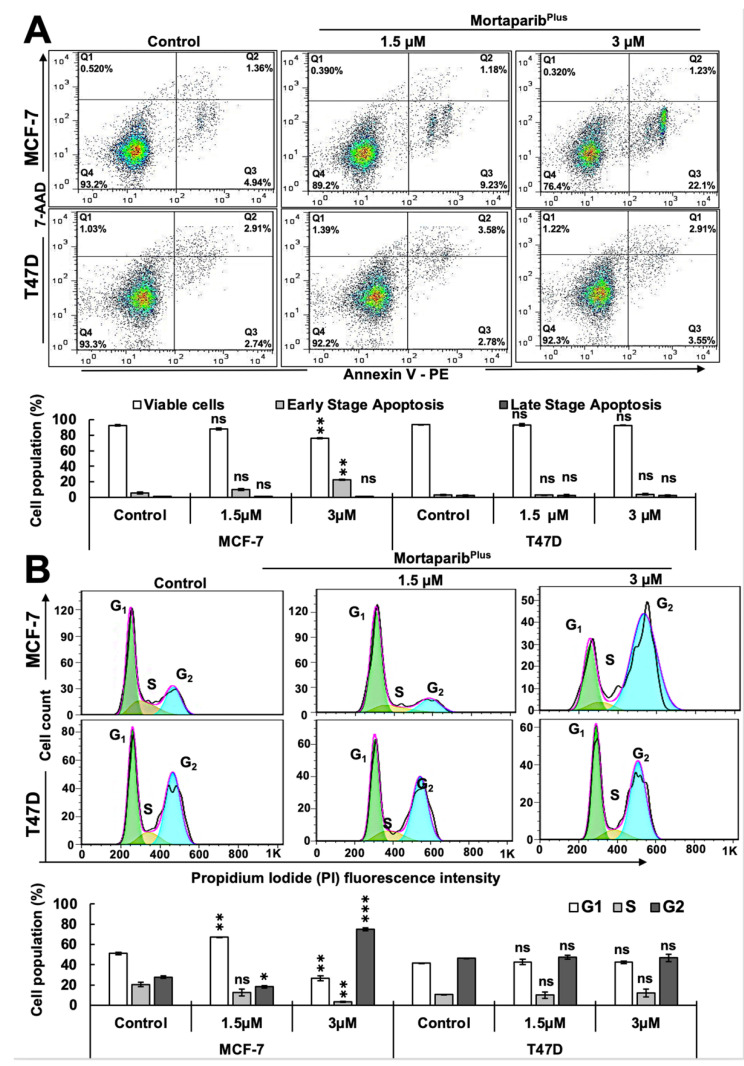
Mortaparib^Plus^ induced apoptosis and G_2_/M cell cycle arrest only in the wild type p53- harboring Luminal-A breast cancer cells. Flow cytometric analyses for control and 48 h Mortaparib^Plus^-treated cells showed an increase in the early apoptotic cell populations in Mortaparib^Plus^-treated MCF-7, but not T47D, cells (**A**). Using the same flow cytometric platform, cell cycle progression analyses for control and 48 h-Mortaparib^Plus^-treated cells revealed that Mortaparib^Plus^ (3 μM) induced a strong G_2_/M cell cycle arrest in MCF-7 cells; Mortaparib^Plus^-treated T47D cells were not arrested at either G_1_/S or G_2_/M cell cycle phases (**B**). The quantified data represent mean ± SD obtained from independent biological replicates; *p*-values were calculated using unpaired Student’s *t*-test. ns > 0.05, * ≤0.05, ** ≤0.01, and *** ≤0.001 represent non-significant, significant, very significant, and highly significant, respectively.

In order to further investigate whether the Mortaparib^Plus^-mediated activation of wild-type p53 function in MCF-7 cells could effectively initiate p53-mediated tumor suppression cell fates, we examined apoptosis initiation and cell cycle progression in control and treated cells by flow cytometry. As shown in Figure 4A, Mortaparib^Plus^-treated MCF-7 cells showed an increase in early apoptotic cell population in a dose-dependent manner. However, Mortaparib^Plus^-treated T47D cells did not show such an increase in the early apoptotic cell population as compared to the untreated control. Furthermore, as shown in Figure 4B, cell cycle analyses revealed that Mortaparib^Plus^ treatment at 1.5 and 3.0 μM doses caused ~20% and ~60% increase in MCF-7 cells at G_1_ and G_2_ phases, respectively. On the other hand, T47D cells were not arrested at either G_1_/S or G_2_/M cell cycle phases. Taken together with the previous molecular results, these data could provide evidence for the reactivation of wild-type p53 signaling in MCF-7 (p53^WT^) cells causing dose-dependent arrest at G_1_/S or G_2_/M phases of cell cycle and/or apoptosis. Mortaparib^Plus^ treated T47D (p53^L194F^) cells did not show these effects.

### 2.3. Mortaparib^Plus^ Induced Hyperactivation of PARP1s in T47D Cells

**Figure 5:**
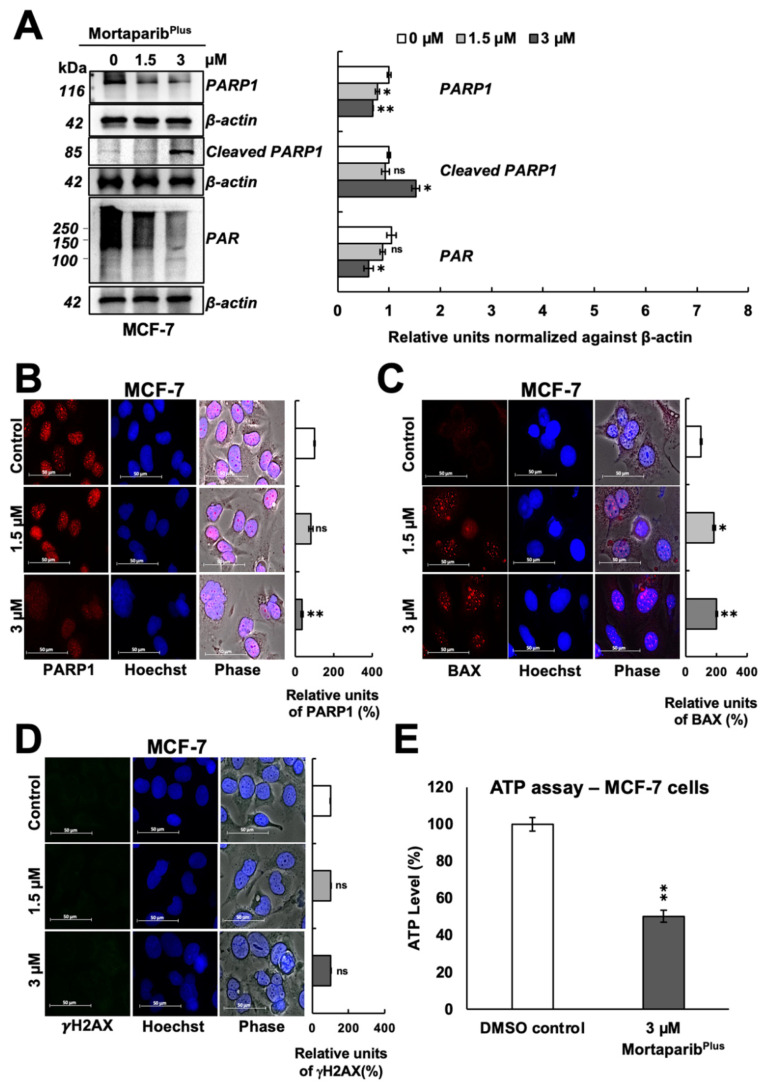
Mortaparib^Plus^-treated Luminal-A breast cancer cells (MCF-7 (p53^WT^)) showed the molecular hallmarks of apoptosis. The levels of the full-length PARP1, the cleaved 89-kDa fragment of PARP1 and PAR polymer were analyzed in control and 48 h-Mortaparib^Plus^-treated MCF-7 cells by Western blotting (**A**). The levels of the full-length PARP1, BAX and the phosphorylated H2A histone variant X (γH2AX) proteins were measured by immunocytochemistry (**B**–**D**). A decrease in the levels of the full-length PARP1 and PAR polymer were observed in Mortaparib^Plus^-treated cells as compared to the untreated control. An increase in the 89-kDa cleaved fragment of PARP1 and BAX protein was observed in the Mortaparib^Plus^-treated MCF-7 cells if compared to the untreated control. Total cellular ATP concentrations in DMSO control and Mortaparib^Plus^-treated MCF-7 cells were measured using a luminescent ATP detection method (**E**). ATP levels were decreased in Mortaparib^Plus^-treated MCF-7 cells compared to the control untreated cells. Scale bar: 50 μm. Full uncropped blots for the Western blotting experiments are shown in Supplementary Figures S15 and S16. The quantified data represents mean ± SD obtained from independent biological replicates; *p*-values were calculated using unpaired Student’s *t*-test. ns > 0.05, * ≤0.05 and ** ≤0.01 represent non-significant, significant, and very significant, respectively.

Apoptosis is a programmed process of cell death with distinctive molecular changes that converge to the activation of a caspases-specific proteolytic cascade. PARP1 cleavage and subsequent inactivation with the decreased levels of the cellular Adenosine Triphosphate (ATP) are prominent molecular hallmarks of apoptosis [35,36]. As p53 signaling was activated in Mortaparib^Plus^-treated MCF-7 cells resulting in initiation of apoptosis (Figure 4A), we observed a decrease in the expression of the full-length PARP1 as measured by Western blotting (Figure 5A) and supported by immunocytochemistry (Figure 5B).

**Figure 6:**
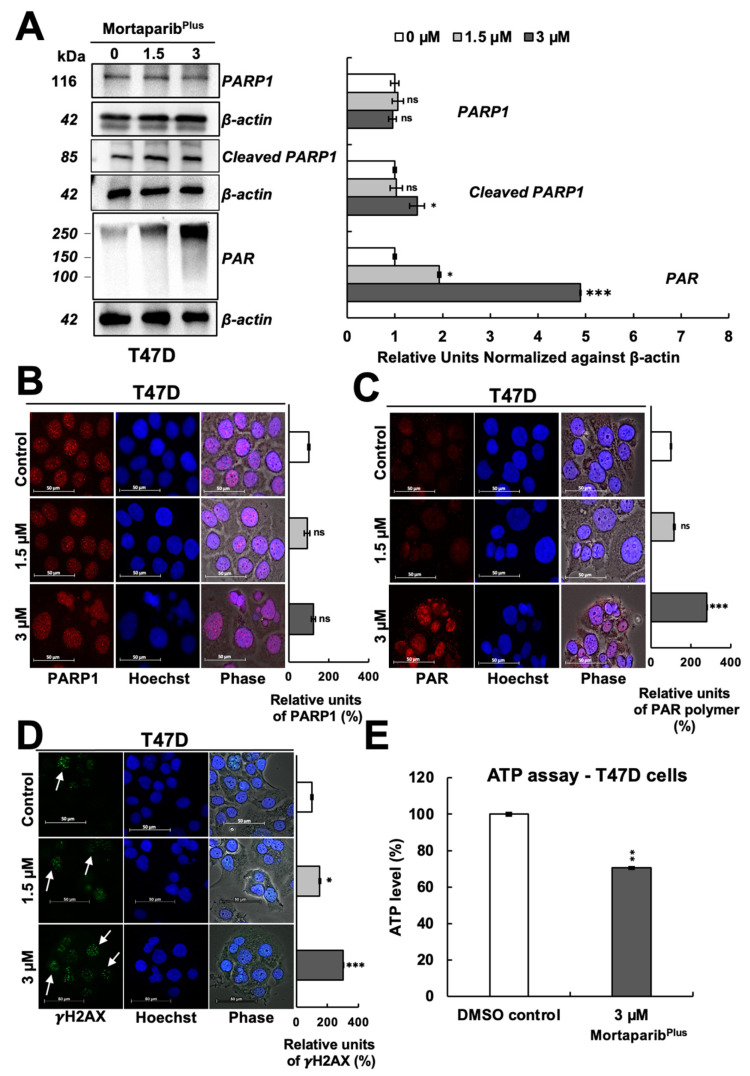
Mortaparib^Plus^ caused an accumulation of PAR polymer and DNA damage in T47D (p53^L194F^) cells. The levels of the full-length PARP1, the cleaved 89-kDa fragment of PARP1 and PAR polymer were analyzed in control and 48 h Mortaparib^Plus^-treated cells by Western blotting (**A**). The levels of the full-length PARP1 protein, PAR polymer and the phosphorylated H2A histone variant X (γH2AX) protein were measured by immunocytochemistry (**B**–**D**). No significant change in the levels of the full-length PARP1 was observed in Mortaparib^Plus^-treated T47D cells as compared to the untreated control. A significant accumulation of PAR polymer and the foci of the phosphorylated H2A histone variant X (γH2AX) were observed in the Mortaparib^Plus^-treated T47D cells as compared to the untreated control. Total cellular ATP concentrations in DMSO control and Mortaparib^Plus^-treated T47D cells were measured using a luminescent ATP detection method (**E**). ATP levels were decreased in Mortaparib^Plus^-treated T47D cells compared to the DMSO control-treated cells. Full uncropped blots for the Western blotting experiments are shown in Supplementary Figures S17 and S18. Scale bar: 50 μm. The quantified data represent mean ± SD obtained from independent biological replicates; *p*-values were calculated using unpaired Student’s *t*-test. ns > 0.05, * <0.05, ** <0.01 and *** <0.001 represent non-significant, significant, very significant, and highly significant, respectively.

Furthermore, as shown in Figure 5A, a decrease in the levels of PAR polymer was detected with an increase in the 89-kDa cleaved fragment of PARP1 in the Mortaparib^Plus^-treated MCF-7 cells. Additionally, BAX protein (the major regulator of the p53-mediated apoptosis) showed an increase in the Mortaparib^Plus^-treated MCF-7 cells as measured by immunocytochemistry (Figure 5C). As shown in Figure 5E, ATP levels were decreased in Mortaparib^Plus^-treated MCF-7 cells compared to the untreated control cells. Such molecular changes in treated MCF-7 cells are typical biomarkers for the establishment of apoptosis as a cellular death fate [36,37]. On the other hand, as apoptosis was not initiated in the treated T47D cells (Figure 4A), the levels of PARP1 remained unchanged (Figure 6A,B). However, we interestingly observed a profound increase in the levels of PAR polymer in Mortaparib^Plus^-treated T47D cells, both by Western blotting (Figure 6A) and immunocytochemistry (Figure 6C). We predicted that such an increase in PAR polymer could be due to the undue PARP1 catalytic activity resulting from the excessive increase in the burden of DNA damage that could be found by the immunostaining of the phosphorylated H2A histone variant X (γH2AX). As shown in Figure 6D, we indeed found a remarkable increase in γH2AX foci in the Mortaparib^Plus^-treated T47D cells as compared to the untreated control. Such an increase was not observed in the Mortaparib^Plus^-treated MCF-7 cells (Figure 5D). On the other hand, the latter showed a remarkable increase in ROS in Mortaparib^Plus^ treated (at 3 μM) cells (Supplementary Figure S3) and marked by the rapid induction of apoptosis (Figure 4A). Of note, the increase in ROS was not observed either in T47D or the normal fibroblasts (Supplementary Figure S3). Independent Western blotting and immunocytochemistry assays also revealed a lack of change in the levels of γH2AX between control and Mortaparib^Plus^-treated TIG-3 and MRC-5 normal lung fibroblasts (Supplementary Figure S4). Taken together, these data supported the idea that the accumulation of DNA damage that did not trigger either the growth arrest or apoptosis, was unique in Mortaparib^Plus^-treated T47D cells.

**Figure 7:**
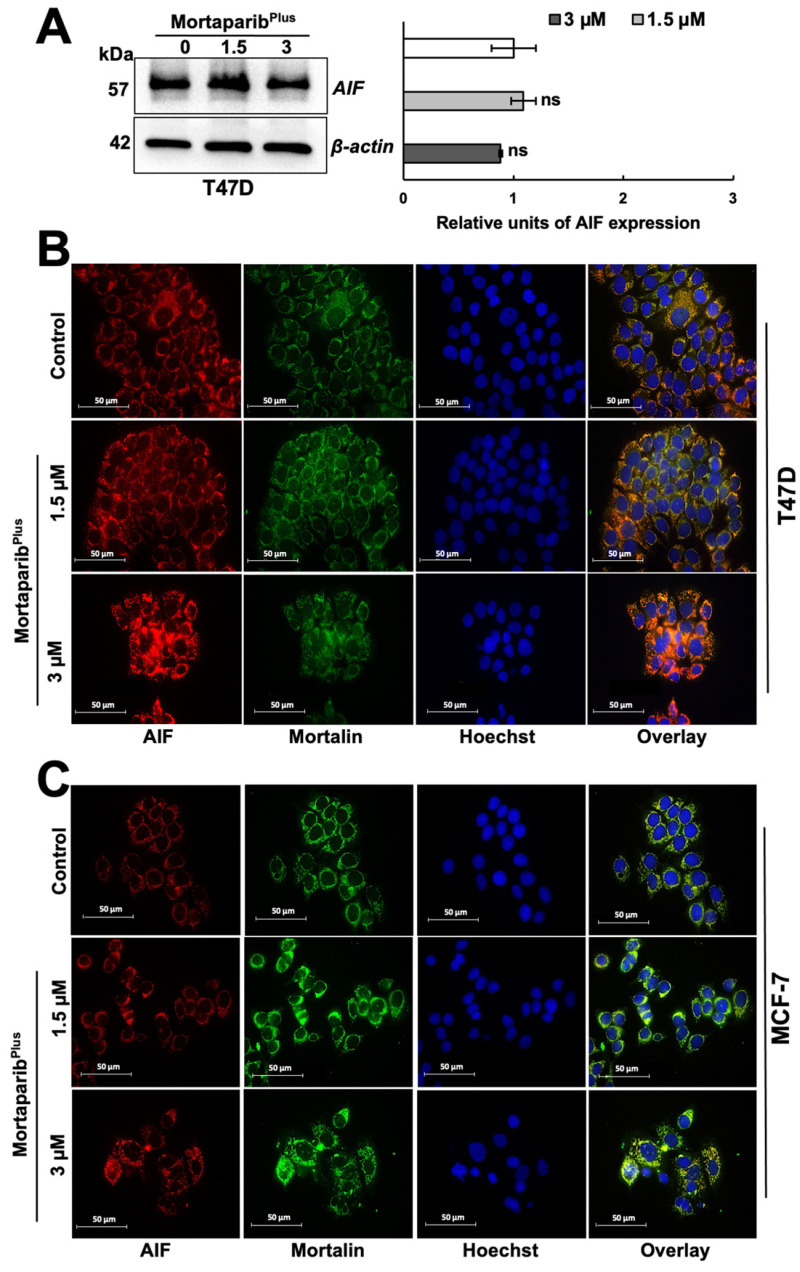
Mortaparib^Plus^ treated cells did not show translocation of apoptosis-inducing factor (AIF) to the nuclei. AIF’s expression level in control and 48 h Mortaparib^Plus^-treated T47D cells was examined by Western blotting (**A**). There were no significant changes in AIF’s expression levels in control and Mortaparib^Plus^-treated T47D cells. AIF’s subcellular localization in control and 48 h-Mortaparib^Plus^-treated T47D and MCF-7 cells was analyzed by immunocytochemistry. Co-localization of mortalin and AIF was observed in T47D cells (**B**). MCF-7 cells showed a low level of expression of AIF as compared to T47D cells; its colocalization with mortalin was observed and more prominent in Mortaparib^Plus^-treated cells (**C**). Full uncropped blots for the Western blotting experiments are shown in Supplementary Figure S18. Scale bar: 50 mm. The quantified data represent mean ± SD obtained from independent biological replicates; *p*-values were calculated using unpaired Student’s *t*-test. ns > 0.05 represents non-significant.

**Figure 8:**
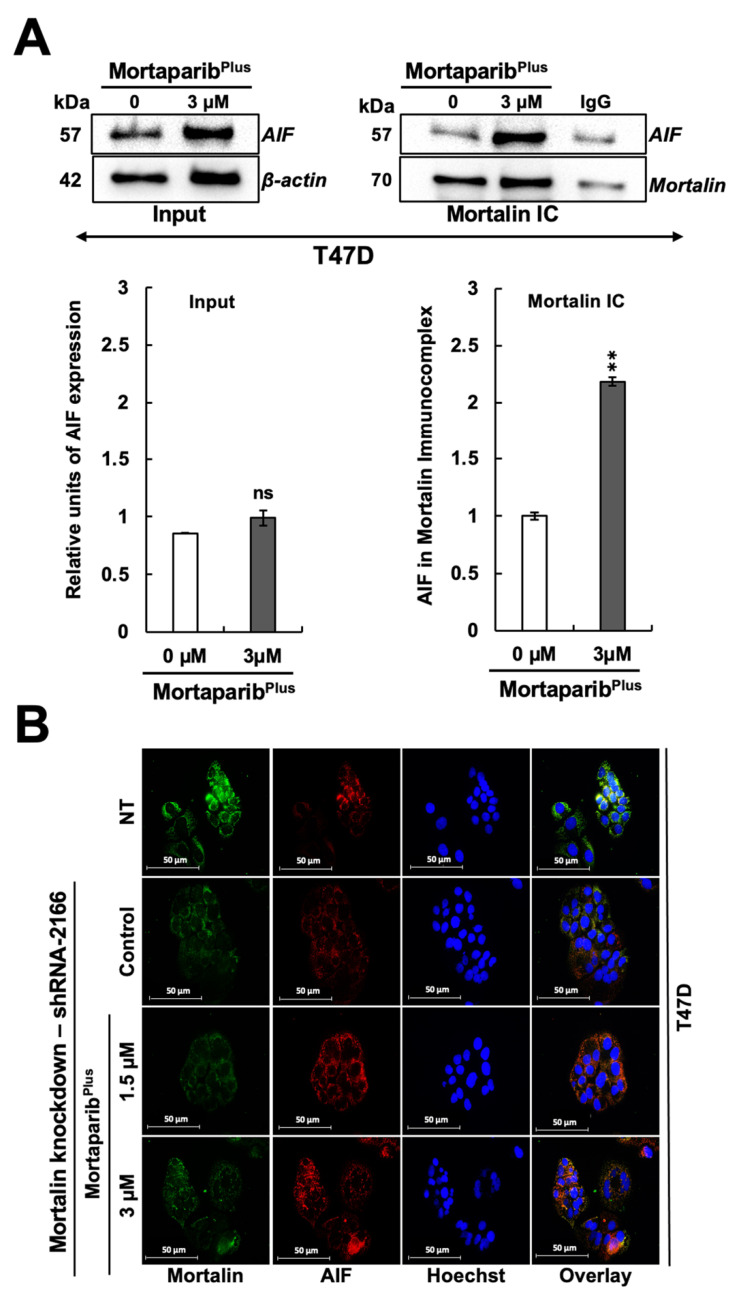
Mortaparib^Plus^ treatment did not abrogate AIF-mortalin interaction in T47D (p53^L194F^). AIF–mortalin interaction in both control and 48 h Mortaparib^Plus^-treated T47D cells were analyzed by co-immunoprecipitation (**A**). An increase in AIF fractions was observed in the mortalin complexes immunoprecipitated from Mortaparib^Plus^-treated T47D cells as compared to both the untreated and IgG controls. Mortalin knockdown could not relocate AIF to the nuclei of T47D cells (p53^L194F^). Mortalin and AIF subcellular localizations were examined by immunocytochemistry after mortalin knockdown using a mortalin-specific shRNA (shRNA2166) (**B**). Mortalin knockdown was not sufficient to liberate AIF from mortalin complex even after the Mortaparib^Plus^-induced PAR accumulation. NT represents Non-Transfected cells. Full uncropped blots for the Co-IP experiments are shown in Supplementary Figure S19. Scale bar: 50 mm. The quantified data represent mean ± SD obtained from independent biological replicates; *p*-values were calculated using unpaired Student’s *t*-test. ns > 0.05 and ** ≤0.01 represents non-significant and very significant.

PAR polymer has been reported as a death signal molecule [38], and its accumulation is considered as a hallmark for PAR-Thanatos or PARP1-mediated cell death [37]. As PAR-Thanatos is mainly characterized by the cellular depletion of ATP levels and translocation of the mitochondrial apoptosis-inducing factor (AIF) to the nucleus for inducing chromatin condensation and DNA degradation [39], we examined the ATP levels and both AIF expression and subcellular localization in control and Mortaparib^Plus^-treated T47D cells. As shown in Figure 6E, although apoptosis was not initiated in the Mortaparib^Plus^-treated T47D cells, total cellular ATP levels showed a decrease as compared to the control vehicle-treated cells. We next examined the AIF levels in the control and Mortaparib^Plus^-treated T47D cells and found that there was no significant change in AIF’s expression level in the treated T47D cells (Figure 7A). Interestingly, as shown in Figure 7B, AIF was not translocated to the nucleus after the Mortaparib^Plus^-mediated PAR accumulation in T47D cells. As previously reported [40,41,42,43], mortalin hinders the translocation of AIF to the nucleus (as shown in Figure 7B, AIF and mortalin colocalization is marked by yellow co-staining in the cytoplasm). These data revealed that in spite of PAR accumulation and the decrease in ATP levels, AIF was not translocated to the nucleus in Mortaparib^Plus^-treated T47D cells (Figure 7B). MCF-7, as compared to T47D, cells showed a lower level of AIF protein. However, Mortaparib^Plus^ treatment caused an increase in AIF (Figure 7C). As shown in Figure 8A, co-immunoprecipitation of mortalin and AIF from control and Mortaparib^Plus^-treated T47D cell lysates revealed an increase in AIF in mortalin immunocomplexes in the later. These data suggested that Mortaparib^Plus^ could not abrogate the interaction between mortalin and AIF confirming the mortalin-mediated cytoplasmic sequestration of AIF in control and Mortaparib^Plus^-treated T47D cells. As shown in Figure 2C, Mortaparib^Plus^-treated T47D cells showed a small increase in mortalin expression as compared to the untreated cells. Since such an increase in mortalin levels could potentially be implicated in AIF’s cytoplasmic sequestration, we next examined if mortalin knockdowns by mortalin specific shRNA (shRNA2166) could relocate AIF to the nucleus in these cells. As shown in Figure 8B, mortalin knockdown was not enough to liberate AIF from mortalin complex even after the Mortaparib^Plus^-induced PAR accumulation.

This could be due to the strong cytoplasmic sequestration of AIF by multiple interaction partners from the HSP70 family of proteins other than mortalin [41]. Taken together, we found that Mortaparib^Plus^ causes abrogation of mortalin–p53 complexes and transcriptional reactivation of wild-type p53, but not the mutant p53^L194F^. In T47D cells, Mortaparib^Plus^ induced PARP1 hyperactivation, as supported by accumulation of PAR and decrease in ATP levels. However, T47D cells escaped PAR-Thanatos due to the cytoplasmic sequestration of AIF attributed to its interaction with mortalin (and possibly other HSP70 family proteins).

## 3. Discussion

Both immunohistochemistry (IHC)- and gene expression-based classifications of breast cancers define the luminal-A subtype to have ER-positive, PR-positive or -negative (PR > 20%, according to the 2013’s St. Gallen updates [44]), HER2-negative and low level of the proliferation marker Ki-67 (Ki-67 < 14%, according to the 2013’s St. Gallen updates [44]). Luminal-A breast cancers are highly sensitive to anti-estrogen endocrine therapies. However, two major characteristics made it a relatively unmet medical need and warranted further endeavors to identify personalized precision treatments. Firstly, it often exhibits an intrinsic or acquired resistance to anti-estrogen therapies. Not only mis-sense mutations and/or amplifications in the Estrogen Receptor*-α* gene sequence (ESR1), but other regulators had also been linked to the endocrine therapy resistance; for instance, miRNAs had been previously reported as mediators of anti-estrogen resistance [1]. Secondly, according to the recent work by Pawan Poudel et al. [33], it has been shown to possess a highly heterogenous nature with almost five hetero-cellular subtypes (Stem-like, Enterocyte, Goblet-like, Inflammatory, and Transit-amplifying Luminal-A breast cancer) [33].

p53 acts as a sensor for many stress signals including oncogenic signaling pathway activation. After the oncogenic activation-dependent post-translational modification of p53, it acts as a sequence-specific transcription factor to transactivate a wide array of genes responsible for the major tumor suppression programs (apoptosis, cell cycle arrest and cell senescence). This could be attributed to the fact that p53 is inactivated in more that 50% of all human tumors. p53 inactivation could be due to a mis-sense mutation in the DNA binding domain sequence, rapid degradation by the overexpression of MDM2 and/or deletion or epigenetic silencing of p14^ARF^, and the cytoplasmic sequestration through binding to the HSP70/GRP75/mortalin [45]. In the present study, based on the information that p53 mutations are not common in estrogen-responsive tumors (only 20% in breast cancers) [46], we tried to reactivate the p53 signaling through its liberation from mortalin–p53 complex shuttle freely to the nucleus to execute its tumor-suppression duties as a transcription factor. Previously, a screening system was established using MCF-7 cells to visually detect the reactivation of the p53 canonical signaling (p53 nuclear translocation) associated with the hallmark of this class of molecules (the shift in mortalin staining from perinuclear to pan-cytoplasmic). In four rounds of screenings, 4-[(1E)-2-(2-phenylindol-3-yl)-1-azavinyl]-1,2,4-triazole (Mortaparib^Plus^) was identified [31]. Ideally, a small molecule activating the p53 signaling should induce differential molecular effects and/or cellular fates in the wild-type, mutant- and null-p53 cell lines within the same disease model [47,48]. However, based on the status of p53 in cancer cell lines deposited in the International Agency for Research on Cancer TP53 database, there are only wild-type p53 cell lines (MCF-7, HCC1428, HCC712 and ZR-75-1) and mutant p53 cell lines (EFM-19, T47D, BT485, CAMA-1 and MDA-MB-415) within the Luminal-A breast cancer disease model [32]. Hence, to address our query about the possible molecular mechanism of Mortaparib^Plus^, we chose MCF-7 and T47D cancer cell lines to represent the Luminal-A breast cancer, and MRC-5 and TIG-3 to represent the normal non-tumorigenic cell model.

In the MTT cytotoxicity assays, where the cell viability is estimated by the conversion of the yellow MTT to the purple formazan crystals by cells’ mitochondrial dehydrogenases, low doses of Mortaparib^Plus^ showed a dose-dependent cytotoxicity against both Luminal-A breast cancer cell lines; the normal lung fibroblasts were relatively resistant to the same doses. Such primary viability results enabled us to define the Luminal-A cancer cell-specific toxicity range (1*–*3 μM). However, the viabilities of both Mortaparib^Plus^-treated cancer cell lines were reduced in a dose-dependent manner; phenotypically, only MCF-7 cells (p53^WT^) showed stress signs. Hence, additional non-REDOX dependent cytotoxicity assays (Crystal Violet; CV and Lactate Dehydrogenase; LDH) were performed to overcome the MTT assay-specific false-positive results that could have been generated as a consequence of particular condition in which cellular metabolism is affected. As shown in Figure 1B and Figure S1, in CV and LDH assays, Mortaparib^Plus^ showed a dose-dependent cytotoxicity preferentially to MCF-7 cells (p53^WT^); T47D cells (p53^L194F^) responded similar to the normal lung fibroblasts. Of note, computational structural homology analyses revealed no similarities between Mortaparib^Plus^ and the known chemotherapeutic or endocrine anticancer drugs, suggesting that Mortaparib^Plus^ may possess a unique molecular mode of action. At a molecular level, although Mortaparib^Plus^ abrogated mortalin–p53 interactions in both MCF-7 and T47D cells, it activated the p53 signaling only in MCF-7 cells that showed (i) nuclear enrichment of p53, (ii) reactivation of transcriptional reactivation function of p53 and (iii) the transactivation of p53 downstream target gene (*p21^WAF1^)* responsible for tumor suppression functions (cell cycle arrest, cell senescence and apoptosis) of p53. This Mortaparib^Plus^-mediated differential activation of the p53 signaling was proved to be mediated, or at least enhanced, through the liberation of p53 from the mortalin complexes as follows: (i) the change in mortalin staining pattern from perinuclear to pan-cytoplasmic and (ii) the reduced p53 fractions in immunoprecipitated mortalin complexes. Interestingly, the Mortaparib^Plus^-mediated activation of p53 signaling was sufficient to induce apoptosis as a p53-dependent tumor suppression fate in MCF-7 (p53^WT^), but not in T47D (p53^L194F^), cells. The data were also supported by cell cycle analysis that showed dose-dependent G_1_/S and G_2_/M arrest in MCF-7, but not T47D, cells. In T47D cells, due to a mutation in the p53 region involved in its binding to DNA, the p53-dependant transcriptional activation-driven apoptotic cascade was disabled. Indeed, Mortaparib^Plus^-treated T47D cells did not undergo apoptosis, rather showed an increase in the burden of DNA damage as seen by the increased level of *γ*H2AX foci. This was also associated with the accumulation of PAR polymer signifying hyperactivation of PARP1, as well as a decrease in ATP. However, these molecular changes did not result in PAR-Thanatos (a PARP1-mediated form of cell death) due to the inability of the mitochondrial AIF to translocate to the nucleus to induce chromatinolysis and DNA degradation. Taken together, these data supported the capability of Mortaparib^Plus^ to cause unique p53-dependent (transcriptional activation driven) and -independent (PARP 1 driven) anticancer activity. However, it was not capable of abrogating AIF-mortalin complexes in T47D cells. Therefore, it is predictive that the cancer cells possessing enriched levels of such complexes may not respond strongly to Mortaparib^Plus^. Taken together, we report multimodal anticancer potential of Mortaparib^Plus^ that warrant further attention in laboratory and clinical studies.

## 4. Materials and Methods

### 4.1. Cell Culture and Drug Treatment

Luminal-A breast cancer cell line (MCF-7) and normal lung fibroblast (MRC-5) and (TIG-3) cells were obtained from the Japanese Cancer Research Resources Bank (JCRB, Japan). Luminal-A breast cancer cell line (T47D) was purchased from the DS Pharma Biomedical Co. Ltd., Japan. All cell lines were cultured and maintained in Dulbecco’s Modified Eagle’s Medium (DMEM)—low glucose with L-glutamine and phenol red (FUJI FILM Wako Pure Chemical Corporation, Osaka, Japan) supplemented with 5% fetal bovine serum to make the complete culture medium and incubated in a humidified incubator (37 °C and 5% CO_2_) (PHC Corporation). Breast cancer cells were estrogen-deprived to conceal its previously reported effect on p53 signaling [46]. 4-[(1E)-2-(2-phenylindol-3-yl)-1-azavinyl]-1,2,4-triazole (Mortaparib^Plus^) (molecular weight: 287.32 g/mol) was purchased from NAMIKI SHOJI Co., Ltd. (Shinjuku, Japan). Working concentrations were freshly prepared in the complete cell culture medium before each experiment.

### 4.2. MTT Cytotoxicity Assay

Cytotoxicity testing of Mortaparib^Plus^ against both breast cancer cells and normal lung fibroblasts was performed through the quantitative colorimetric MTT assay. In this method, the cell viability is estimated by the conversion of the yellow 3-(4,5-dimethylthiazol-2-yl)-2,5-diphenyltetrazolium bromide salt (MTT) (Sigma Aldrich, Meguro, Japan) to the purple formazan crystals by cells’ mitochondrial dehydrogenases. Cells were seeded at an approximate density of 3000–4000 cells per well in 96-well plates and cultured for 24 h in a humidified incubator (37 °C and 5% CO_2_) to attach and recover from the harvest stress. Then, five working concentrations of Mortaparib^Plus^ (1 μM, 2 μM, 3 μM, 4 μM and 5 μM) were freshly prepared in the complete culture medium. The next day, cells were treated with these concentrations (at least three technical replicas for each concentration). After 48 h incubation period, MTT (0.5 mg/mL) was added to the cell culture medium and the incubation time was extended for another 4 h. MTT-containing-cell culture medium was replaced with 100 μL of Dimethyl Sulfoxide (DMSO), and plates were shaken for 5 min to completely dissolve the formed formazan crystals. The optical density was measured at 570 nm using a spectrophotometer (Tecan infinite M200^®^ Pro, Tecan, Mannedorf, Switzerland). Finally, the percentage viability related to the DMSO vehicle control was measured using the following equation [49]:

(1) $$\%\;\mathrm{Cell}\;\mathrm{Viability}\;\mathrm{related}\;\mathrm{to}\;\mathrm{DMSO}\;\mathrm{control}=[\;\frac{OD\;treated\;cells-OD\;media\;blank}{OD\;DMSO\;control-OD\;media\;blank\;}\;]\;\times \;100$$

Optical Denisty (OD) for treated cells: value of the mean absorbance readings for cells exposed to Mortaparib^Plus^.

Optical Denisty (OD) for DMSO control: value of the mean absorbance readings for cells exposed to the maximum concentration of the vehicle (DMSO) in the complete culture medium.

Optical Density (OD) for media blank: the value of the mean absorbance readings for the media minus cells.

### 4.3. Crystal Violet Assay

The cytotoxicity of Mortaparib^Plus^ was determined in breast cancer cells and normal lung fibroblasts by Crystal Violet assay (Abcam (ab232855)). Cells (3000–4000 cells/well) were plated in 96-well plate, allowed to settle overnight and treated with varying doses of control (DMSO) and Mortaparib^Plus^ (1–5 µM) for 48 h. Time course assay was performed with 3 µM Mortaparib^Plus^ for 24–96 h. Crystal violet assay was performed according to the manufacturer’s protocol, followed by measurement of optical density at 595 nm using a microplate reader (Tecan infinite M200^®^ Pro, Tecan, Mannedorf, Switzerland).

### 4.4. Lactate Dehydrogenase (LDH) Cytotoxicity Assay

Cells (3000–4000 cells/well) were plated in 96-well plate. After settlement, cells were treated with varying doses of Mortaparib^Plus^ (1 µM, 2 µM, 3 µM, 4 µM and 5 µM) and incubated for 48 h in a humidified incubator (37 °C and 5% CO_2_). Cellular cytotoxicity was represented by measuring the extracellular lactate dehydrogenase (LDH) quantitatively in breast cancer cells and normal lung fibroblasts using a colorimetric assay with Thermo Scientific Pierce LDH Cytotoxicity Assay Kit (Thermo Scientific, Rockford, IL, USA). The assay was performed according to the manufacturer’s instructions.

### 4.5. Phase Contrast Light Microscopy

Cells were cultured in control and Mortaparib^Plus^-supplemented complete culture medium in cell culture dishes (TPP, Trasadingen, Switzerland). After 48 h incubation time, and with the aim to examine possible Mortaparib^Plus^-induced stress phenotypes, images, for both breast cancer cells and normal lung fibroblasts, were captured under phase contrast light microscope (Nikon TS100-F, Tokyo, Japan).

### 4.6. Western Blotting (WB)

Control and Mortaparib^Plus^-treated cells were harvested by trypsinization and centrifugation. Then, cells pellets were PBS-washed and lysed using RIPA lysis buffer (Thermo Fisher Scientific Inc., Rockford, IL, USA) supplemented with a complete protease inhibitor cocktail (complete, MiniTM) (Roche Applied Science, Mannheim, Germany). Cell lysates were centrifuged at 15,000 rpm for 15 min, and then, the supernatant containing soluble proteins was collected for protein quantification. The protein concentrations were measured by Bi-Cinchonic Acid (BCA) assay (Thermo Fisher Scientific). Equal amounts of protein (20*–*40 μg) were separated in 8*–*12% SDS-Poly Acrylamide Gel Electrophoresis (SDS-PAGE) and transferred to a polyvinylidene difluoride (PVDF) membrane (Millipore, Billerica, MA, USA) using a semidry transfer blotter (ATTO Corporation, Japan) or a wet transfer blotter (Bio-Rad, Hercules, CA, USA.). Membranes were blocked with 3% Bovine Serum Albumin Fraction-V (FUJIFILM Wako Pure Chemical Corporation, Osaka, Japan) at room temperature for 2 h. Blocked membranes were probed at 4 °C (overnight) with the following target-specific primary antibodies: Anti-mortalin (37*-*6) raised in our lab; anti-p53 [DO-1] (sc-126), anti-BAX [N-20] (sc-493), and anti-PARP1 [F-2] (sc-8007) from Santa Cruz Biotechnology (Dallas, TX, USA.); anti-PUMA (D30C10), and anti-p21 (12D1) from Cell Signaling Technology (Danvers, MA, USA.); anti-Poly ADP-Ribose polymer [10H] (ab14459), and anti-cleaved PARP1 (ab4830) from Abcam (Cambridge, UK). After washing with TBS-T, the blots were incubated for one hour with the following secondary antibodies conjugated to horseradish peroxidase: anti-rabbit IgG and anti-mouse IgG (Santa Cruz Biotechnology, USA). Then, after another TBS-T wash cycle, blots were developed through the enhanced chemiluminescence reaction (ECL) (GE Healthcare, Chicago, IL, USA). β-actin antibody (Abcam, UK) was used as an internal loading control. ImageJ 1.46 software (NIH, Bethesda, MD, USA) was used for the quantification of the quantitated luminescent signals.

### 4.7. Immunocytochemistry (ICC)

Cells were seeded on 18 mm glass coverslips placed in 12-well culture plates (TPP^®^, Trasadingen, Switzerland) with a seeding density of 40,000 cells per well and cultured for 24 h in a humidified incubator (37 °C and 5% CO_2_) to allow attachment and recovery from the harvest stress. Then, cells were treated with Mortaparib^Plus^ for 48 h followed by washing with PBS and fixation in pre-chilled methanol:acetone (1:1) at 4 °C (10 min). After that, the fixation solution was removed, and cells were washed with PBS and permeabilized by PBS-T for 10 min on a slow shaker. After permeabilization, the glass coverslips were blocked with 2% bovine serum albumin in PBS-T for 1 h. Then, coverslips were incubated overnight at 4 °C with the following primary antibodies: anti-mortalin (C-13) raised in our lab; anti-p53 [FL-393] (sc-6243), anti-BAX [N-20] (sc-493), and anti-AIF [E-1] (sc-13116), anti-PARP1 [F-2] (sc-8007) antibodies from Santa Cruz Biotechnology (TX, USA.); anti-γH2AX (Ser139) antibody (20E3) from Cell Signaling Technology (MA, USA.), and anti-Poly ADP-Ribose polymer [10H] (ab14459) from Abcam (Cambridge, U.K.). Protein localization and expression levels were visualized by secondary staining with Alexa Flour-488 conjugated goat anti-rabbit IgG (Catalogue#A-11034), Alexa Flour-488 conjugated goat anti-mouse (Catalogue#A-11001), R-Phycoerythrin conjugated Goat anti-mouse IgG1 (Catalogue#*p*-21129) or Alexa Flour-546 conjugated Goat anti-rabbit (Catalogue#A-11035) antibodies (Molecular Probes, Eugene, OR, USA) based on the species in which the primary antibody was raised. After the incubation with secondary antibody, cells were washed with PBS-T for 10 min followed by nuclear counter-staining with Hoechst 33342 (Molecular Probes). Then, cells were washed with the following: one time with PBS-T for 10 min, one time with PBS for 10 min and one time with Milli-Q H_2_O for 10 min, respectively. Finally, cells were mounted in FA mounting solution (VMRD, Inc., Pullman, WA, USA) and examined using a Zeiss Axiovert 200 M immunofluorescence microscope and analyzed by AxioVision 4.6 software (Carl Zeiss, Oberkochen, Germany).

### 4.8. Luciferase Reporter Assay

Cells were seeded in 6-well culture plates (TPP, Trasadingen, Switzerland) with a seeding density of 300,000 cells per well and cultured for 48 h in a humidified incubator (37 °C and 5% CO_2_) to be allowed to attach, recover from the harvest stress and grow until 80% confluency. Then, cells were transiently transfected (3 μg per well) with the previously isolated PG13-Luc (wild type p53 consensus sequence binding sites) plasmid DNA using X-tremeGENE HP DNA Transfection Reagent (Roche Applied Science, Mannheim, Germany) in a volume ratio of 1:1 plasmid DNA to transfection reagent in serum-free DMEM medium. After an overnight transfection, the transfection serum-free media were replaced with complete culture media, and cells were incubated for 24 h in humidified incubator to recover from the transfection stress. Then, cells were treated with Mortaparib^Plus^ for 48 h, after which whole-cell lysates were prepared in passive lysis buffer (PLB) (PLB; Cat. #E1941, Promega, Madison, WI, USA.). The Luciferase activity was estimated by using the Luciferase Reporter Assay system (Promega, WI, USA) and a luminescent plate reader (Tecan infinite M200^®^ Pro, Tecan, Mannedorf, Switzerland).

### 4.9. Apoptosis Analysis

Cells were seeded in 50 mm cell culture dishes (TPP, Trasadingen, Switzerland) with a seeding density of 400,000 cells per well and cultured for 24 h followed by treatment with Mortaparib^Plus^ for 24 h. After that, cells were harvested along with the treatment media and centrifuged for 1200 rpm for 2 min to collect both cells and possible apoptotic bodies. Finally, 100 μL of single-cell suspension was mixed with 100 μL Guava Nexin reagent (4500-0450) (Luminex Corporation, Austin, TX, USA) and incubated in dark for 20 min (note: cell concentration was 1 × 10^6^ cell per sample). Finally, the mixture (cell suspension and Nexin reagent) was acquired using Guava PCA-96 Flow Cytometry machine (Luminex Corporation, Austin, TX, USA). FlowJoTM software Version 7.6, Flow Jo, LLC, Ashland, OR, USA) was used for the analysis of the obtained flow cytometry data.

### 4.10. Cell Cycle Analysis

Cells were seeded in 50 mm cell culture dishes (TPP, Trasadingen, Switzerland) with a seeding density of 400,000 cells per well and cultured for 24 h followed by treatment with Mortaparib^Plus^ for 24 h. Then, MCF-7 cells and T47D cells were treated with Mortaparib^Plus^ for 24 h. Control and treated cells were harvested by trypsin-EDTA, washed with cold PBS, fixed with 70% ethanol on slow vortex and kept at −20 °C for 48 h. The fixed cells were centrifuged at 3000 rpm at 4 °C for 10 min followed by two cycles of cold PBS washing. Then, cells were stained with Guava Cell Cycle Reagent (4500-0220) (Luminex Corporation, Austin, TX, USA.) in dark for 30 min. RNA was degraded by treatment with RNase-A (1 mg/mL, −37 °C, 30 min) and analyzed using Guava PCA-96 System (Luminex Corporation). FlowJo*^TM^* software (Version 7.6, Flow Jo, LLC, Ashland, OR, USA) was used for the analysis of the obtained flow cytometry data.

### 4.11. ATP Assay

Total cellular ATP concentration in control and Mortaparib^Plus^-treated cells was determined using a Luminescent ATP Detection Assay Kit (ab113849) (Abcam, Cambridge, UK) following the manufacturer’s protocol.

### 4.12. ROS Assay

Cells (40,000/well) were cultured on a glass coverslip in 12-well plates, allowed to settle overnight and treated with 1.5 μM and 3 μM Mortaparib^Plus^ for 48 h. Image-IT^TM^ LIVE green Reactive Oxygen Species detection kit (Molecular Probes, Eugene, OR, USA) was used for ROS detection, following the manufacturer’s recommendations. tBHP was used as a positive control. Zeiss Axiovert 200 M Microscope (Carl Zeiss, Tokyo, Japan) was used for capturing the Images and analysis by AxioVision 4.6 software (Carl Zeiss, Tokyo, Japan).

### 4.13. Immunoprecipitation

Control and Mortaparib^Plus^-treated cells were harvested, PBS-washed, and lysed using NP40 lysis buffer. Cell lysates containing 300*–*500 μg protein from control and Mortaparib^Plus^-treated cells were incubated with anti-mortalin polyclonal antibody raised in our lab or control normal rabbit IgG (2729) (Cell Signaling Technology, MA, USA) at 4 °C overnight in slow rotation. Fifty microliters of resuspended Protein A/G PLUS-Agarose beads (sc-2003) (Santa Cruz Biotechnology, USA) was added to the mixture and incubated for 1*–*4 h. Immunoprecipitants were collected by centrifugation at 2500 rpm at 4 °C for 5 min. Pellets were washed with NP-40 lysis buffer followed by centrifugation at 2500 rpm at 4 °C for 5 min 5*–*6 times. Then, immunoprecipitants were boiled in SDS sample buffer, resolved on SDS-PAGE and subjected to the routine Western blotting analysis with anti-p53 mouse monoclonal antibody (DO-1) and anti-AIF [E-1] (sc-13116) from Santa Cruz Biotechnology (USA.).

### 4.14. Mortalin Knockdown

Mortalin-specific shRNA plasmid “2166-shRNA” was previously constructed through the insertion of oligonucleotides corresponding to the mortalin gene (GenBank NM_004134) (sequence: 5′-GCCAGAAGGACAACATATGTTCAAGAGACATATGTTGTCCTTCTG GCTTTTTTGGAAA-3′) into the BamHI and HindIII sites of pSilencer 2.1-U6 neo vector (Ambion, Austin, TX, USA.) [20]. T47D cells were seeded into a 6-well plate (200,000 cells/well) and incubated to attach and recover from the harvest stress. Then, T47D cells in each well were transiently transfected with 4 μg of mortalin shRNA-2166 using the X-tremeGENE HP DNA transfection reagent and incubated for 24 h. After that, cells were harvested and seeded again on 18 mm glass coverslips placed in 12-well culture plates. After recovery, cells were treated with Mortaparib^Plus^ for 24 h. Finally, mortalin and AIF subcellular localizations were checked using immunocytochemistry (ICC) as previously described.

### 4.15. Statistical Analysis

Unpaired *t*-test (GraphPad Prism online calculator) has been performed to determine the degree of significance between the control and experimental samples from three or more independent experiments. Statistical significance was defined as significant (* *p*-value ≤ 0.05), very significant (** *p*-value ≤ 0.01), highly significant (*** *p*-value ≤ 0.001) and extremely significant (**** *p*-value ≤ 0.0001).

## 5. Conclusions

In Luminal-A breast cancer in vitro models, Mortaparib^Plus^ blocked the interaction of mortalin with p53 irrespective of its status (wild type or mutant). In the wild-type p53 harboring Luminal-A breast cancer model (MCF-7), it reactivated its transcriptional activation functions resulting in the induction of growth arrest and apoptosis. In the mutant p53^L194F^-harboring cells (T47D), Mortaparib^Plus^ could induce a decrease in ATP, accumulation of DNA damage and hyperactivation of PARP1 (PAR accumulation). However, it did not yield either apoptosis or PAR-Thanatos due to its inability to abrogate AIF-mortalin complexes in T47D cells. Therefore, cancer cells possessing enriched levels of such complexes are predicted to refract to its anticancer activity. Mortaparib^Plus^ possesses multi-modal anticancer activity and warrants further experimental and clinical investigations.

## Data Availability

All datasets used and/or analyzed during the current study are available in the manuscript and Supplementary Information Files.

## Supplementary Materials

The following are available online at https://www.mdpi.com/article/10.3390/cancers13123043/s1, Figure S1: Cytotoxicity analyses lactate dehydrogenase (LDH) assays of TIG-3, MRC-5, MCF-7 and T47D cells after 48 h of treatment with either DMSO (control) or Mortaparib^Plus^ (A) and Cytotoxicity analyses Crystal Violet (CV) assay of T47D cell after 24 h, 48 h, 72 h and 96 h of treatment with either DMSO (control) or Mortaparib^Plus^ (B). The quantitation of the data represents mean ± SD, obtained from independent biological replicates, is shown. *p*-values were calculated using unpaired Student’s *t*-test. * ≤0.05, ** ≤0.01 and *** ≤0.001 represent significant, very significant and highly significant, respectively. Figure S2: Identification of Mortaparib^Plus^ as a novel small molecule. Structural homology of Mortaparib^Plus^ with several known chemotherapeutic drugs (A) and anti-estrogen therapeutic drugs (B) clinically used for Luminal-A breast cancer treatment is shown. Figure S3: Reactive Oxygen Species (ROS) detection in control and 48 h-Mortaparib^Plus^ -treated MCF-7, T47D and MRC-5 cells. Tert-Butyl hydroperoxide (tBHP) was used as a positive control. Figure S4: Western blots and Immunocytostaining images to detect γH2AX in control and 48 h Mortaparib^Plus^-treated MRC-5 and TIG-3 cells. β-actin was used as an internal loading control. Figure S5. Western blots (full-uncropped) and reading density number of the band for the protein of interest (p53) detected from both mortalin immunocomplexes and input of Mortaparib^Plus^-treated and control MCF-7 cell lysates (Figure 2A). For the immunoprecipitated samples, mortalin bands were used to normalize equal immunocomplexes. For the input samples, β-actin was used as an internal loading control. Figure S6. Western blots (full-uncropped) and reading density number of the band for the protein of interest (p53) detected from both mortalin immunocomplexes and input of Mortaparib^Plus^-treated and control T47D cell lysates (Figure 2B). For the immunoprecipitated samples, mortalin bands were used to normalize an equal immunocomplexes. For the input samples, β-actin was used as an internal loading control. Figure S7. Western (full uncropped) blots and reading density number of the band for the protein of interest (p53 and mortalin) detected from Mortaparib^Plus^-treated and control MCF-7 cell lysates (Figure 2C). β-actin was used as an internal loading control. Figure S8. Western blots (full-uncropped) and reading density number of the band for the protein of interest (p21 and PUMA) detected from Mortaparib^Plus^-treated and control MCF-7 cell lysates (Figure 2C). β-actin was used as an internal loading control. Figure S9. Western blots (full-uncropped) and reading density number of the band for the protein of interest (BAX) detected from Mortaparib^Plus^-treated and control MCF-7 cell lysates (Figure 2C). β-actin band was used as an internal loading control. Figure S10. Western blots (full-uncropped) and reading density number of the band for the protein of interest (p53 and mortalin) detected from Mortaparib^Plus^-treated and control T47D cell lysates (Figure 2C). β-actin was used as an internal loading control. Figure S11. Western blots (full-uncropped) and reading density number of the band for the protein of interest (p21 and PUMA) detected from Mortaparib^Plus^-treated and control T47D cell lysates (Figure 2C). β-actin was used as an internal loading control. Figure S12. Western blots (full-uncropped) and reading density number of the band for the protein of interest (BAX) detected from Mortaparib^Plus^-treated and control T47D cell lysates (Figure 2C). β-actin was used as an internal loading control. Figure S13. Western blots (full-uncropped) and reading density number of the band for the protein of interest (p53 and p21) detected from Mortaparib^Plus^-treated and control TIG-3 cell lysates (Figure 2D). β-actin was used as an internal loading control. Figure S14. Western blots (full-uncropped) and reading density number of the band for the protein of interest (p53 and p21) detected from Mortaparib^Plus^-treated and control MRC-5 cell lysates (Figure 2D). β-actin was used as an internal loading control. Figure S15. Western blots (full-uncropped) and reading density number of the band for PARP1 and cleaved PARP1 detected from Mortaparib^Plus^-treated and control MCF-7 cell lysates (Figure 5A). β-actin was used as an internal loading control. Figure S16. Western blots (full-uncropped) and reading density number of the band for PAR detected from Mortaparib^Plus^-treated and control MCF-7 cell lysates (Figure 5A). β-actin was used as an internal loading control. Figure S17. Western blots (full-uncropped) and reading density number of the band for PARP1 and cleaved PARP1 detected from Mortaparib^Plus^-treated and control T47D cell lysates (Figure 6A). β-actin was used as an internal loading control. Figure S18. Western blots (full-uncropped) and reading density number of the band for PAR (Figure 6A) and AIF (Figure 7A) detected from Mortaparib^Plus^-treated and control T47D cell lysates. β-actin was used as an internal loading control. Figure S19. Western blots (full-uncropped) and reading density number of the band for the protein of interest (AIF) detected from both mortalin immunocomplexes and inputs of Mortaparib^Plus^-treated and control T47D cell lysates (Figure 8A). For the immunoprecipitated samples, mortalin bands were used to normalize equal immunocomplexes. For the input samples, β-actin was used as an internal loading control.

## Abbreviations

ADP: Adenosine diphosphate; BAX: Bcl-2-associated X protein; BRCA1/2: Breast Cancer susceptibility gene 1/gene 2; CIP1: Cyclin-dependent kinase inhibitor 1; CO2: Carbon Dioxide; ddH2O: Double-distilled H2O; DNA: Deoxyribonucleic Acid; ER-α: Estrogen Receptor alphal; GRP75: Glucose Regulated Protein 75; Hsp70: 70 kDa Heat shock protein; HSPA9: Heat Shock Protein Family A (Hsp70) Member 9; IgG: Immunoglobulin G; kDa: Kilodalton; MCF-7: Michigan Cancer Foundation-7; MDM2: Mouse double minute 2 homolog/ E3 ubiquitin-protein ligase; miRNAs: micro RNAs; mM: Millimeter; MRC-5: Medical Research Council cell strain 5; Mthsp70: Mitochondrial 70kDa Heat shock protein; NAD+: Nicotinamide Adenine Dinucleotide; NP-40: Nonidet P-40; OD: Optical Density; PBS: Phosphate-Buffered Saline; PUMA: p53 upregulated modulator of apoptosis; RNA: Ribonucleic Acid; shRNA: Short hairpin Ribonucleic Acid; TIG-3: Tokyo Institute of Gerontology-3 lung fibroblast; μM: Micromolar.

## References

[1] Szostakowska M., Trębińska-Stryjewska A., Grzybowska E.A., Fabisiewicz A. (2019). Resistance to endocrine therapy in breast cancer: Molecular mechanisms and future goals. Breast Cancer Res. Treat..

[2] Eroles P., Bosch A., Pérez-Fidalgo J.A., Lluch A. (2012). Molecular biology in breast cancer: Intrinsic subtypes and signaling pathways. Cancer Treat. Rev..

[3] Ring A., Dowsett M. (2004). Mechanisms of tamoxifen resistance. Endocrine-Related Cancer.

[4] Horlings H.M., Bergamaschi A., Nordgard S.H., Kim Y.H., Han W., Noh N.-Y., Salari K., Joosse S.A., Reyal F., Lingjaerde O.C. (2008). ESR1 gene amplification in breast cancer: A common phenomenon?. Nat. Genet..

[5] Nielsen K.V., Ejlertsen B., Müller S., Møller S., Rasmussen B.B., Balslev E., Lænkholm A.-V., Christiansen P., Mouridsen H.T. (2010). Amplification of ESR1 may predict resistance to adjuvant tamoxifen in postmenopausal patients with hormone receptor positive breast cancer. Breast Cancer Res. Treat..

[6] Anurag M., Punturi N., Hoog J., Bainbridge M.N., Ellis M.J., Haricharan S. (2018). Comprehensive Profiling of DNA Repair Defects in Breast Cancer Identifies a Novel Class of Endocrine Therapy Resistance Drivers. Clin. Cancer Res..

[7] Wadhwa R., Kaul S., Ikawa Y., Sugimoto Y. (1993). Identification of a novel member of mouse hsp70 family. Its association with cellular mortal phenotype. J. Biol. Chem..

[8] Wadhwa R., Taira K., Kaul S.C. (2002). Mortalin: A potential candidate for biotechnology and biomedicine. Histol. Histopathol..

[9] Wadhwa R., Taira K., Kaul S.C. (2002). An Hsp70 family chaperone, mortalin/mthsp70/PBP74/Grp75: What, when, and where?. Cell Stress Chaperones.

[10] Deocaris C.C., Kaul S.C., Wadhwa R. (2009). The versatile stress protein mortalin as a chaperone therapeutic agent. Protein Pept. Lett..

[11] Kaul S.C., Deocaris C.C., Wadhwa R. (2007). Three faces of mortalin: A housekeeper, guardian and killer. Exp. Gerontol..

[12] Londono C., Osorio C., Gama V., Alzate O. (2012). Mortalin, Apoptosis, and Neurodegeneration. Biomolecules.

[13] Dundas S.R., Lawrie L.C., Rooney P.H., Murray G.I. (2005). Mortalin is over-expressed by colorectal adenocarcinomas and correlates with poor survival. J. Pathol..

[14] Wadhwa R., Takano S., Kaur K., Deocaris C.C., Pereira-Smith O.M., Reddel R.R., Kaul S.C. (2006). Upregulation of mortalin/mthsp70/Grp75 contributes to human carcinogenesis. Int. J. Cancer.

[15] Na Y., Kaul S.C., Ryu J., Lee J.-S., Ahn H.M., Kaul Z., Kalra R.S., Li L., Widodo N., Yun C.-O. (2016). Stress Chaperone Mortalin Contributes to Epithelial-to-Mesenchymal Transition and Cancer Metastasis. Cancer Res..

[16] Yun C.-O., Bhargava P., Na Y., Lee J.-S., Ryu J., Kaul S.C., Wadhwa R. (2017). Relevance of mortalin to cancer cell stemness and cancer therapy. Sci. Rep..

[17] Wadhwa R., Takano S., Robert M., Yoshida A., Nomura H., Reddel R., Mitsui Y., Kaul S.C. (1998). Inactivation of Tumor Suppressor p53 by Mot-2, a hsp70 Family Member. J. Biol. Chem..

[18] Gestl E.E., Böttger S.A. (2012). Cytoplasmic sequestration of the tumor suppressor p53 by a heat shock protein 70 family member, mortalin, in human colorectal adenocarcinoma cell lines. Biochem. Biophys. Res. Commun..

[19] Walker C., Böttger S., Low B. (2006). Mortalin-Based Cytoplasmic Sequestration of p53 in a Nonmammalian Cancer Model. Am. J. Pathol..

[20] Lu W.-J., Lee N.P., Kaul S.C., Lan F., Poon R.T.P., Wadhwa R., Luk J.M. (2011). Mortalin–p53 interaction in cancer cells is stress dependent and constitutes a selective target for cancer therapy. Cell Death Differ..

[21] Grover A., Priyandoko D., Gao R., Shandilya A., Widodo N., Bisaria V.S., Kaul S.C., Wadhwa R., Sundar D. (2012). Withanone binds to mortalin and abrogates mortalin–p53 complex: Computational and experimental evidence. Int. J. Biochem. Cell Biol..

[22] Nigam N., Grover A., Goyal S., Katiyar S., Bhargava P., Wang P.-C., Sundar D., Kaul S.C., Wadhwa R. (2015). Targeting Mortalin by Embelin Causes Activation of Tumor Suppressor p53 and Deactivation of Metastatic Signaling in Human Breast Cancer Cells. PLoS ONE.

[23] Bhargava P., Grover A., Nigam N., Kaul A., Doi M., Ishida Y., Kakuta H., Kaul S.C., Terao K., Wadhwa R. (2018). Anticancer activity of the supercritical extract of Brazilian green propolis and its active component, artepillinï¿½C: Bioinformatics and experimental analyses of its mechanisms of action. Int. J. Oncol..

[24] Widodo N., Kaur K., Shrestha B.G., Takagi Y., Ishii T., Wadhwa R., Kaul S.C. (2007). Selective Killing of Cancer Cells by Leaf Extract of Ashwagandha: Identification of a Tumor-Inhibitory Factor and the First Molecular Insights to Its Effect. Clin. Cancer Res..

[25] Wadhwa R., Sugihara T., Yoshida A., Nomura H., Reddel R., Simpson R., Maruta H., Kaul S.C. (2000). Selective toxicity of MKT-077 to cancer cells is mediated by its binding to the hsp70 family protein mot-2 and reactivation of p53 function. Cancer Res..

[26] Fatokun A.A., Dawson V.L., Dawson T.M. (2014). Parthanatos: Mitochondrial-linked mechanisms and therapeutic opportunities. Br. J. Pharmacol..

[27] Chaudhuri A.R., Nussenzweig A.R.C.A. (2017). The multifaceted roles of PARP1 in DNA repair and chromatin remodelling. Nat. Rev. Mol. Cell Biol..

[28] McCann K.E., Hurvitz S.A. (2018). Advances in the use of PARP inhibitor therapy for breast cancer. Drugs Context.

[29] Abbotts R., Topper M.J., Biondi C., Fontaine D., Goswami R., Stojanovic L., Choi E.Y., McLaughlin L., Kogan A.A., Xia L. (2019). DNA methyltransferase inhibitors induce a BRCAness phenotype that sensitizes NSCLC to PARP inhibitor and ionizing radiation. Proc. Natl. Acad. Sci. USA.

[30] Putri J.F., Bhargava P., Dhanjal J.K., Yaguchi T., Sundar D., Kaul S.C., Wadhwa R. (2019). Mortaparib, a novel dual inhibitor of mortalin and PARP1, is a potential drug candidate for ovarian and cervical cancers. J. Exp. Clin. Cancer Res..

[31] Sari A., Elwakeel A., Dhanjal J., Kumar V., Sundar D., Kaul S., Wadhwa R. (2021). Identification and Characterization of Mortaparib^Plus^—A Novel Triazole Derivative That Targets Mortalin-p53 Interaction and Inhibits Cancer-Cell Proliferation by Wild-Type p53-Dependent and -Independent Mechanisms. Cancers.

[32] Kao J., Salari K., Bocanegra M., Choi Y.-L., Girard L., Gandhi J., Kwei K.A., Hernandez-Boussard T., Wang P., Gazdar A.F. (2009). Molecular Profiling of Breast Cancer Cell Lines Defines Relevant Tumor Models and Provides a Resource for Cancer Gene Discovery. PLoS ONE.

[33] Poudel P., Nyamundanda G., Patil Y., Cheang M.C.U., Sadanandam A. (2019). Heterocellular gene signatures reveal luminal-A breast cancer heterogeneity and differential therapeutic responses. npj Breast Cancer.

[34] Lim L.Y., Vidnovic N., Ellisen L.W., Leong C.-O. (2009). Mutant p53 mediates survival of breast cancer cells. Br. J. Cancer.

[35] Soldani C., Scovassi A.I. (2002). Poly(ADP-ribose) polymerase-1 cleavage during apoptosis: An update. Apoptosis.

[36] Imamura H., Sakamoto S., Yoshida T., Matsui Y., Penuela S., Laird D.W., Mizukami S., Kikuchi K., Kakizuka A. (2020). Single-cell dynamics of pannexin-1-facilitated programmed ATP loss during apoptosis. eLife.

[37] Aredia F., Scovassi A.I. (2014). Poly(ADP-ribose): A signaling molecule in different paradigms of cell death. Biochem. Pharmacol..

[38] Andrabi S.A., Kim N.S., Yu S.-W., Wang H., Koh D.W., Sasaki M., Klaus J.A., Otsuka T., Zhang Z., Koehler R.C. (2006). Poly(ADP-ribose) (PAR) polymer is a death signal. Proc. Natl. Acad. Sci. USA.

[39] Andrabi S.A., Dawson T.M., Dawson V.L. (2008). Mitochondrial and Nuclear Cross Talk in Cell Death. Ann. N. Y. Acad. Sci..

[40] Ravagnan L., Gurbuxani S., Susin S.A., Maisse C., Daugas E., Zamzami N., Mak T., Jäättelä M., Penninger J.M., Garrido C. (2001). Heat-shock protein 70 antagonizes apoptosis-inducing factor. Nat. Cell Biol..

[41] Gurbuxani S., Schmitt E., Cande C., Parcellier A., Hammann A., Daugas E., Kouranti I., Spahr C., Pance A., Kroemer G. (2003). Heat shock protein 70 binding inhibits the nuclear import of apoptosis-inducing factor. Oncogene.

[42] McKeage M.J., Berners-Price S., Galettis P., Bowen R.J., Brouwer W., Ding L., Zhuang L., Baguley B.C. (2000). Role of lipophilicity in determining cellular uptake and antitumour activity of gold phosphine complexes. Cancer Chemother. Pharmacol..

[43] Modak R., Basha J., Bharathy N., Maity K., Mizar P., Bhat A.V., Vasudevan M., Rao V.K., Kok W.K., Natesh N. (2013). Probing p300/CBP Associated Factor (PCAF)-Dependent Pathways with a Small Molecule Inhibitor. ACS Chem. Biol..

[44] Harbeck N., Thomssen C., St. Gnant M. (2013). Gallen 2013: Brief Preliminary Summary of the Consensus Discussion. Breast Care.

[45] Gasco M., Shami S., Crook T. (2002). The p53 pathway in breast cancer. Breast Cancer Res..

[46] Berger C., Qian Y., Chen X. (2013). The p53-Estrogen Receptor Loop in Cancer. Curr. Mol. Med..

[47] Zhang Q., Bergman J., Wiman K.G., Bykov V.J. (2018). Role of Thiol Reactivity for Targeting Mutant p53. Cell Chem. Biol..

[48] Ramraj S.K., Elayapillai S.P., Pelikan R.C., Zhao Y.D., Isingizwe Z.R., Kennedy A.L., Lightfoot S.A., Benbrook D.M. (2020). Novel ovarian cancer maintenance therapy targeted at mortalin and mutant p53. Int. J. Cancer.

[49] Cavanaugh F.P., Moskwa P.S., Donish W.H., Pera P.J., Richardson D., Andrese A.P. (1990). A semi-automated neutral red based chemosensitivity assay for drug screening. Investig. New Drugs.

