# Supplementary material for: Mutant p53L194F Harboring Luminal-A Breast Cancer Cells Are Refractory to Apoptosis and Cell Cycle Arrest in Response to MortaparibPlus, a Multimodal Small Molecule Inhibitor
Source: Cancers (Basel). 2021 Jun 18;13(12):3043. doi: 10.3390/cancers13123043 (PMC8234533; doi:10.3390/cancers13123043)
Supplement: Supplementary file 1 [file cancers-13-03043-s001.zip › cancers-1194348 supplementary.pdf]

cancers

MDPI

Article

Mutant p53^{L194F} Harboring Luminal-A Breast Cancer Cells Are Refractory to Apoptosis and Cell Cycle Arrest in Response to Mortaparib^{Plus}, a Multimodal Small Molecule Inhibitor

Ahmed Elwakeel, Anissa Nofita Sari, Jaspreet Kaur Dhanjal, Hazna Noor Meidinna, Durai Sundar, Sunil C. Kaul, Renu Wadhwa

Supplementary Materials

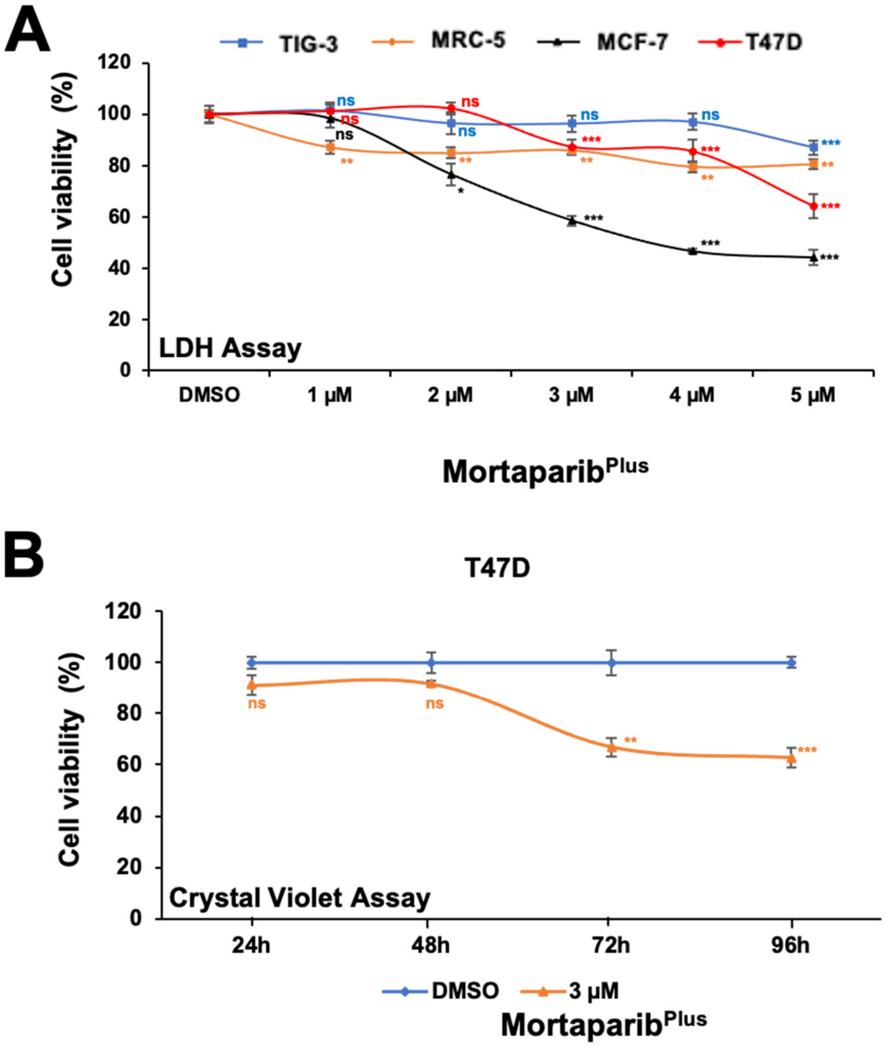

Figure S1. Cytotoxicity analyses Lactate Dehydrogenase (LDH) assays of TIG-3, MRC-5, MCF-7 and T47D cells after 48 h of treatment with either DMSO (control) or Mortaparib^{Plus} (**A**) and cytotoxicity analyses Crystal Violet (CV) assay of T47D cell after 24 h, 48 h, 72 h and 96 h of treatment with either DMSO (control) or Mortaparib^{Plus} (**B**). The quantitation of the data represents mean ± SD, obtained from independent biological replicates, is shown. *p*-values were calculated using unpaired Student's *t*-test. * ≤ 0.05, ** ≤ 0.01 and *** ≤0.001 represent significant, very significant and highly significant, respectively.

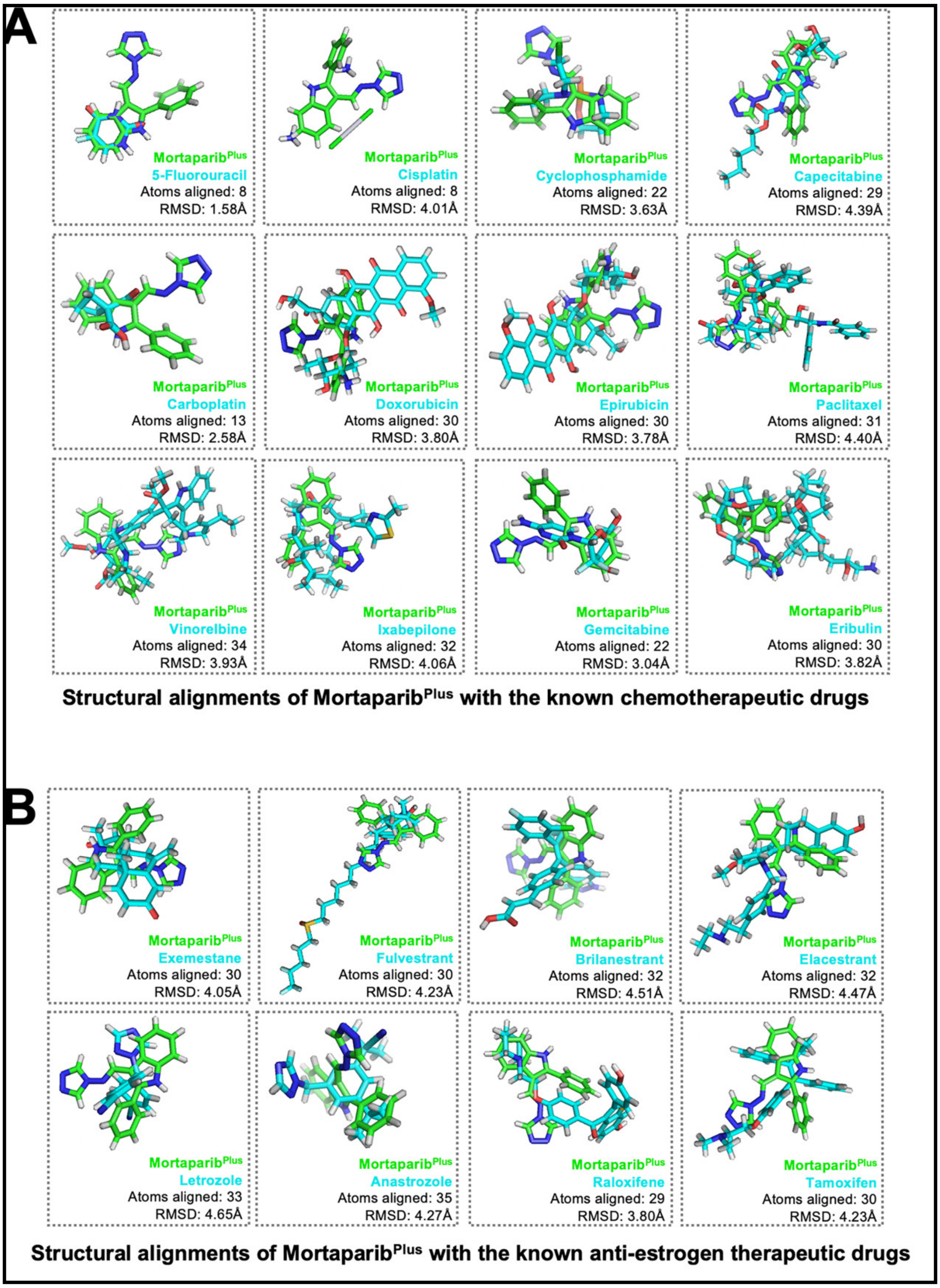

Figure S2. Identification of Mortaparib^{Plus} as a novel small molecule. Structural homology of Mortaparib^{Plus} with several known chemotherapeutic drugs (**A**) and anti-estrogen therapeutic drugs (**B**) clinically used for Luminal-A breast cancer treatment is shown.

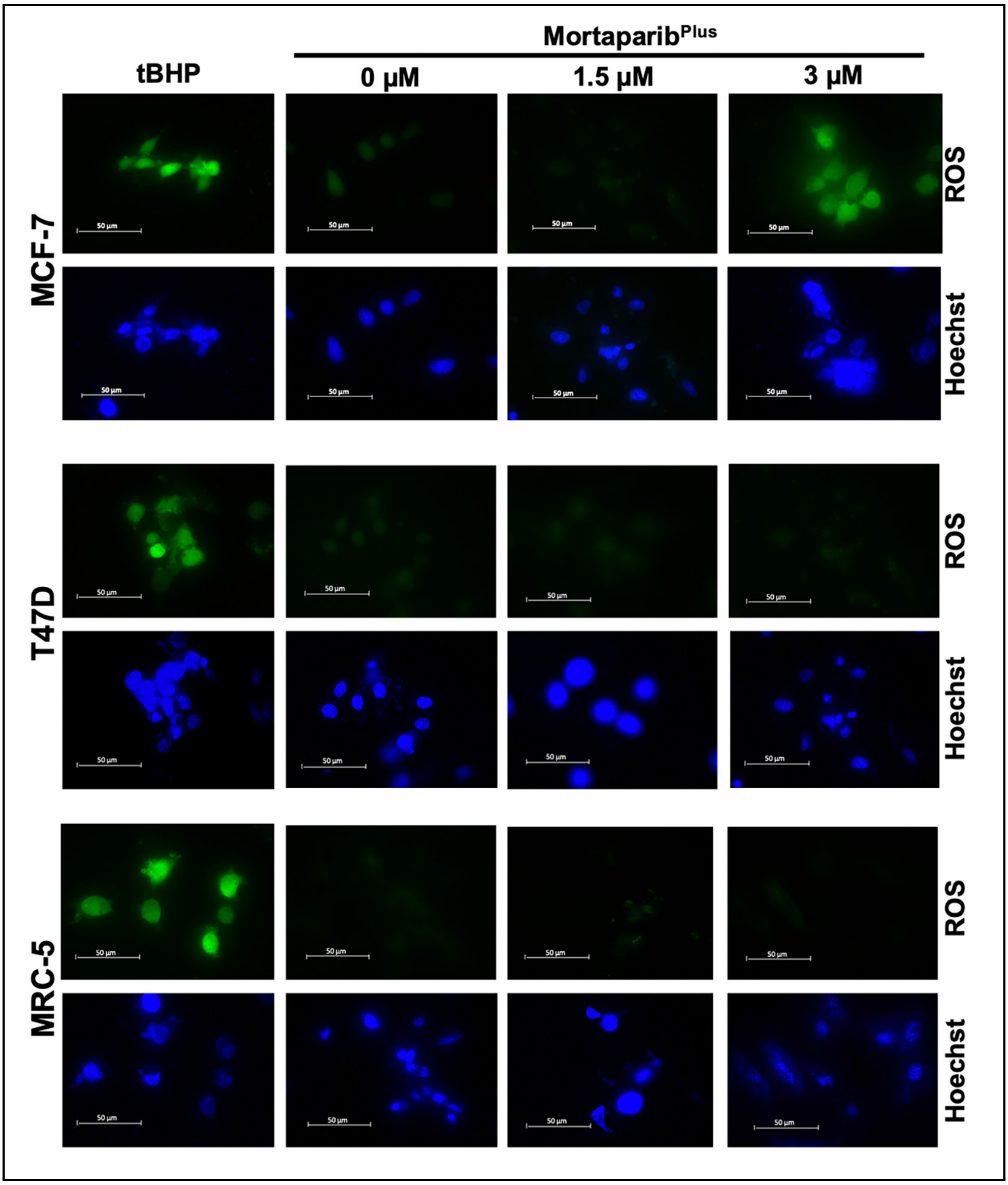

Figure S3. Reactive Oxygen Species (ROS) detection in control and 48 h-Mortaparib^{Plus} -treated MCF-7, T47D and MRC-5 cells. Tert-Butyl hydroperoxide (tBHP) was used as a positive control.

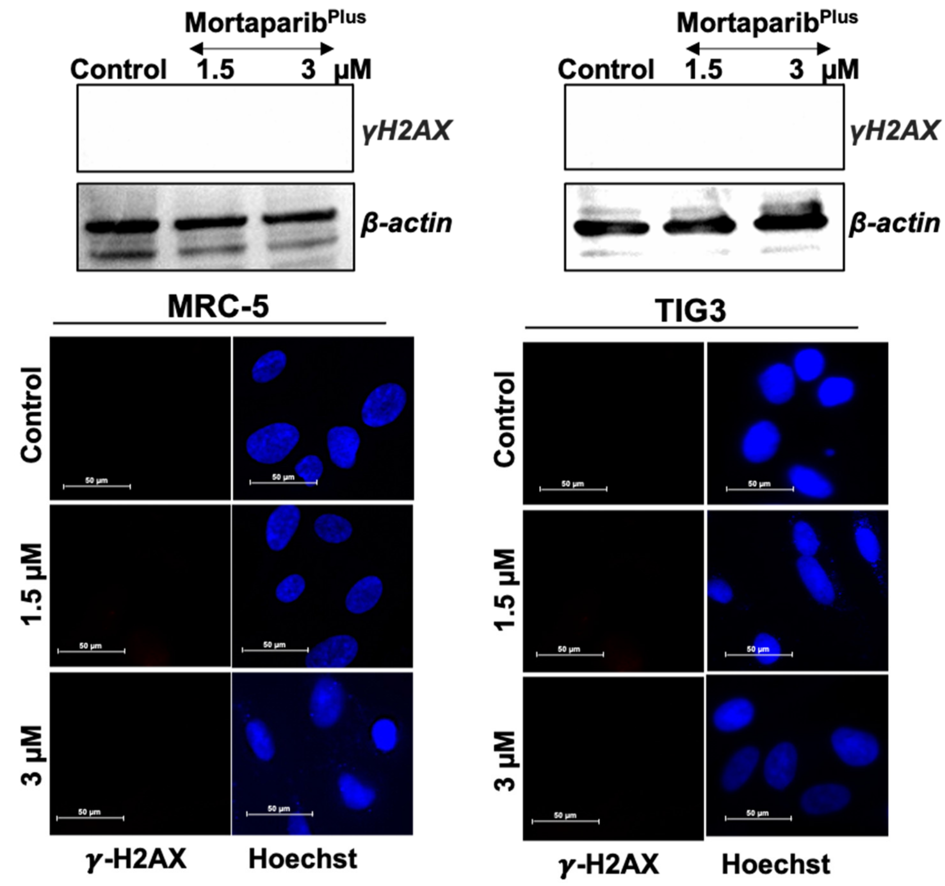

Figure S4. Western blots and Immunostaining images to detect γH2AX in control and 48 h-Mortaparib^{Plus}-treated MRC-5 and TIG-3 cells. β-actin was used as an internal loading control.

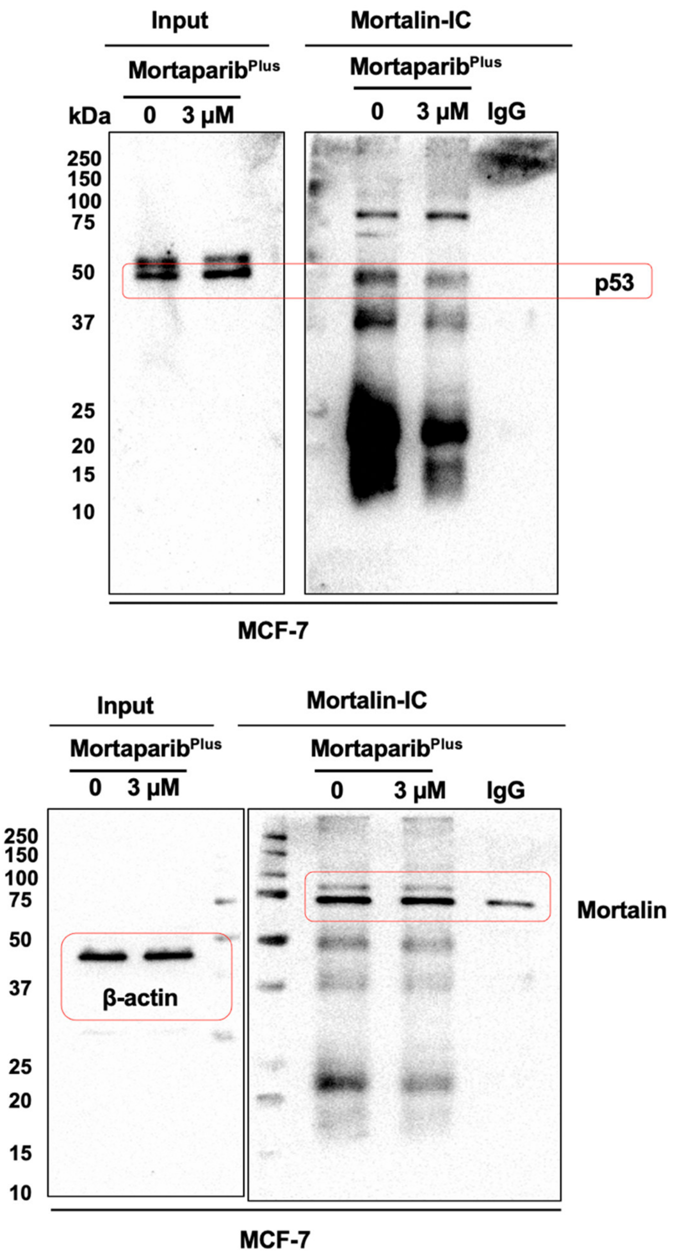

p53	Average of reading density number (Normalized against mortalin band)	fold	SD	p-value
0 μM	0.82700423	1.00000003	0.05954921	
3 μM	0.48956318	0.59197181	0.02491941	0.0178

Figure S5. Western blots (full-uncropped) and reading density number of the band for the protein of interest (p53) detected from both mortalin immunocomplexes and input of Mortaparib^{Plus}-treated and control MCF-7 cell lysates (Figure 2A). For the immunoprecipitated samples, mortalin bands were used to normalize an equal immunocomplexes. For the input samples, β-actin was used as an internal loading control.

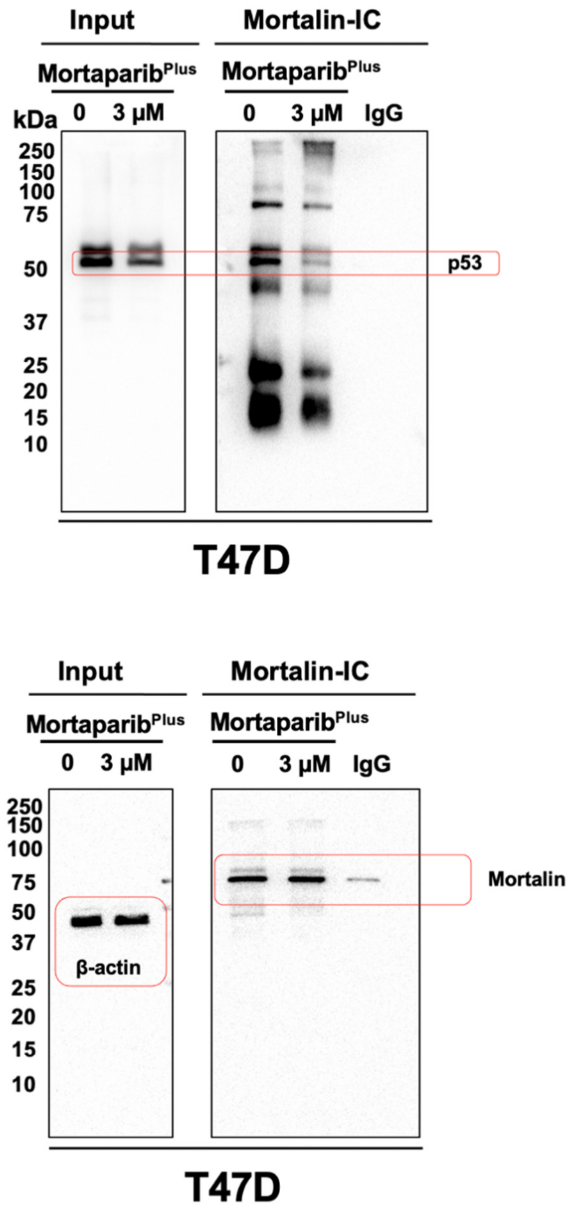

p53	Average of reading density number (Normalized against mortalin band)	fold	SD	p-value
0 μM	0.61230084	0.0139035	1	
3 μM	0.41406492	0.0208047	0.67624425	0.0079

Figure S6. Western blots (full-uncropped) and reading density number of the band for the protein of interest (p53) detected from both mortalin immunocomplexes and input of Mortaparib^{Plus}-treated and control T47D cell lysates (Figure 2B). For the immunoprecipitated samples, mortalin bands were used to normalize an equal immunocomplexes. For the input samples, β-actin was used as an internal loading control.

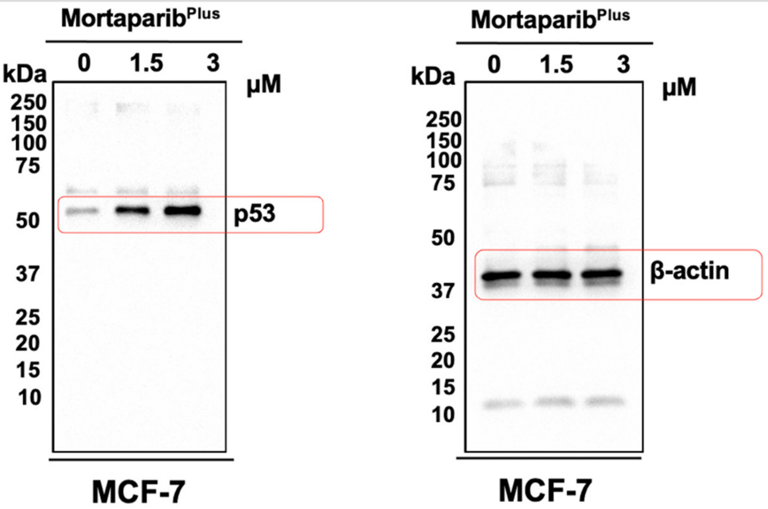

p53 (DO1)	Average of reading density number (Normalized against β-actin band)	SD	Fold change	p value
0 μM	0.10286982	0.02353229	1.000000182	
1.5 μM	0.38875364	0.04638833	3.779084281	0.0020
3 μM	0.62038648	0.02094637	6.030793135	0.0001

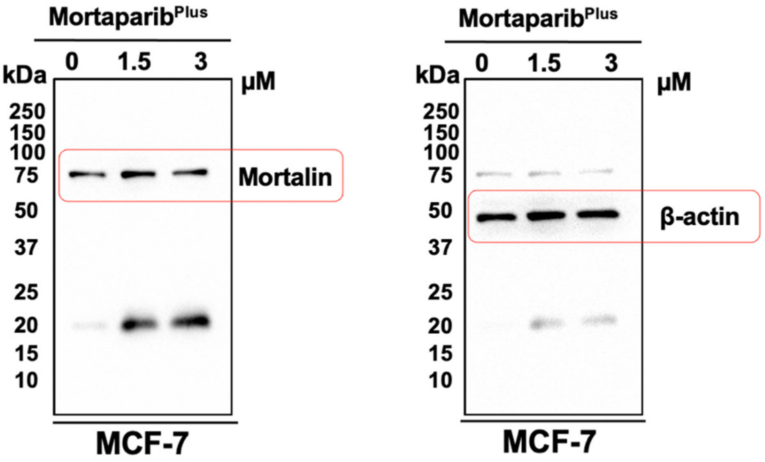

mortalin (37-6)	Average of reading density number (Normalized against β-actin band)	SD	Fold change	p value
0 μM	0.47964038	0.14256573	0.999999948	
1.5 μM	0.5758644	0.09275821	1.200616964	0.3826
3 μM	0.5958997	0.08190425	1.242388455	0.2879

Figure S7. Western blots (full uncropped) and reading density number of the band for the protein of interest (p53 and mortalin) detected from Mortaparib^{Plus}-treated and control MCF-7 cell lysates (Figure 2C). β-actin was used as an internal loading control.

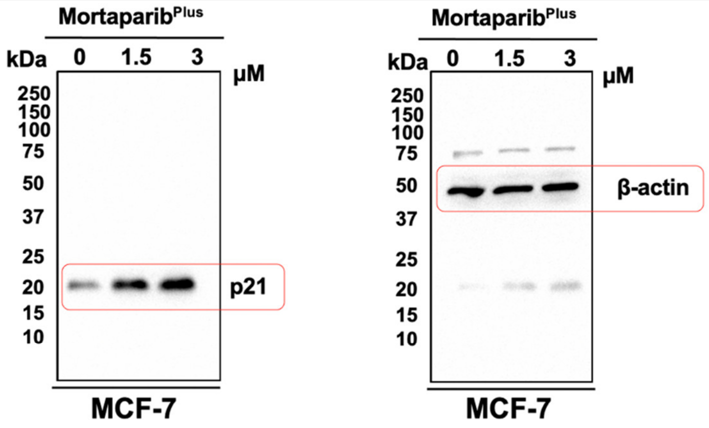

p21	Average of reading density number (Normalized against β-actin band	SD	Fold change	p value
0 μM	0.1644177	0.08907974	0.999999987	
1.5 μM	0.65354117	0.01541571	3.974883323	0.0005
3 μM	0.82514032	0.06119143	5.018561382	0.0004

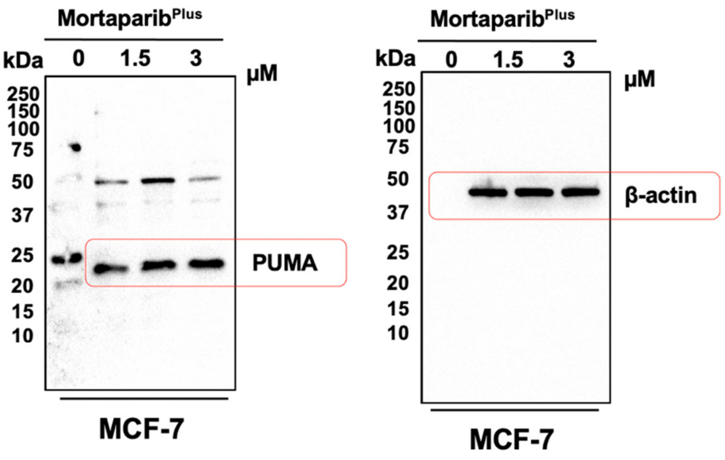

PUMA	Average of reading density number (Normalized against β-actin band	SD	Fold change	p value
0 μM	0.72041	0.02623	1.00001	
1.5 μM	0.78265	0.00959	1.08639	0.0876
3 μM	0.79649	0.01132	1.10561	0.0638

Figure S8. Western blots (full-uncropped) and reading density number of the band for the protein of interest (p21 and PUMA) detected from Mortaparib^{Plus}-treated and control MCF-7 cell lysates (Figure 2C). β-actin was used as an internal loading control.

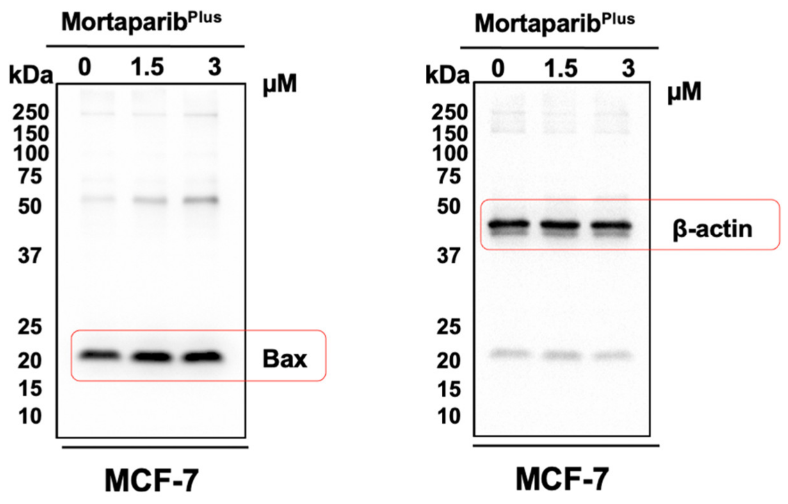

Bax (N-20)	Average of reading density number (Normalized against β-actin band	SD	Fold change	p value
0 µM	0.7012177	0.05152435	1.000000006	
1.5 µM	0.87200888	0.01494211	1.2435637	0.0053
3 µM	0.87186084	0.08227136	1.243352589	0.0382

Figure S9. Western blots (full-uncropped) and reading density number of the band for the protein of interest (BAX) detected from Mortaparib^{Plus}-treated and control MCF-7 cell lysates (Figure 2C). β-actin band was used as an internal loading control.

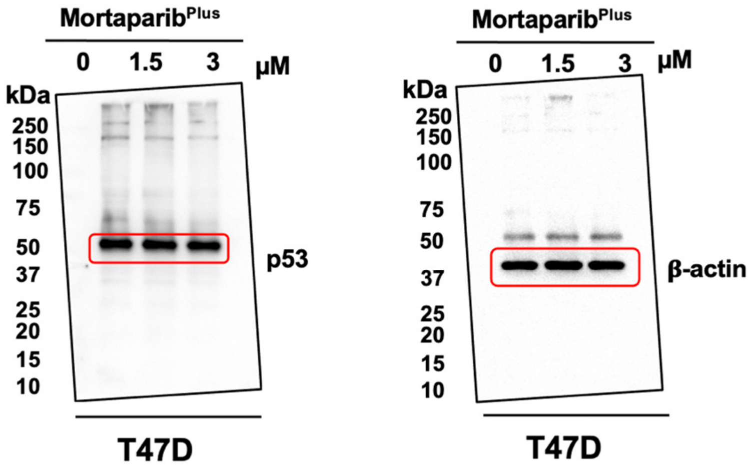

p53 (DO1)	Average of reading density number (Normalized against β-actin band	SD	Fold change	p-value
0 µM	0.88474417	0.14750751	0.99999999	
1.5 µM	0.89241257	0.04843821	1.00866736	0.9507
3 µM	0.8949501	0.03043891	1.01153546	0.9324

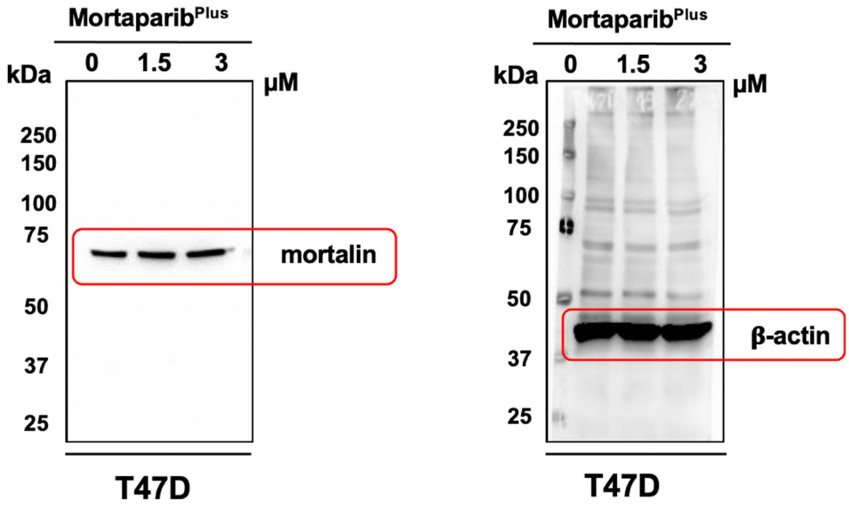

mortalin (37-6)	Average of reading density number (Normalized against β-actin band	SD	Fold change	p-value
0 µM	0.30806124	0.00691385	1.00000079	
1.5 µM	0.40117565	0.00805817	1.30226044	0.0064
3 µM	0.43784967	0.0300053	1.42130835	0.027

Figure S10. Western blots (full-uncropped) and reading density number of the band for the protein of interest (p53 and mortalin) detected from Mortaparib^{Plus}-treated and control T47D cell lysates (Figure 2C). β-actin was used as an internal loading control.

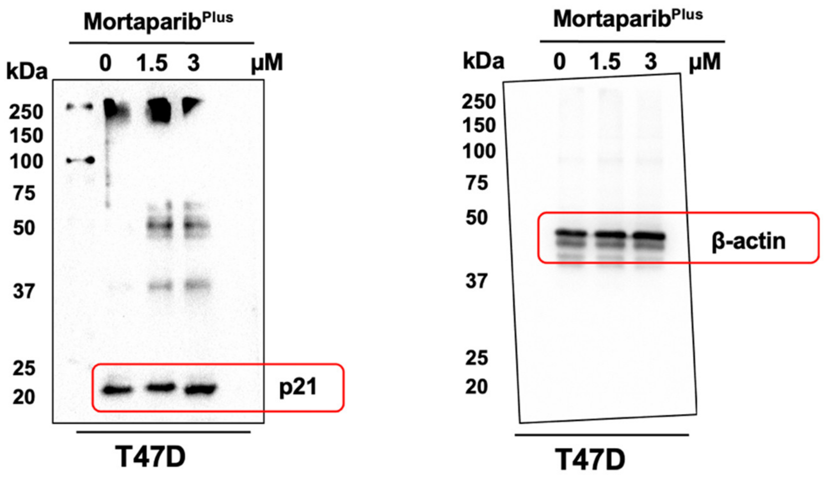

p21	Average of reading density number (Normalized against β-actin band)	SD	Fold change	p-value
0 μM	0.58646693	0.2470145	1.00000005	
1.5 μM	0.62564375	0.22969079	1.06680146	0.824
3 μM	0.72078308	0.2514446	1.22902602	0.4749

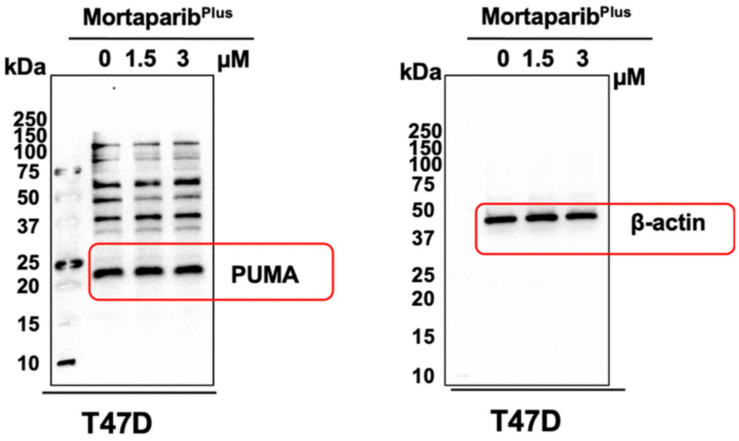

PUMA	Average of reading density number (Normalized against β-actin band)	SD	Fold change	p-value
0 μM	0.82885701	0.0549173	1.00006879	
1.5 μM	0.79416382	0.08521043	0.95820924	0.6762
3 μM	0.73852398	0.06556242	0.89107623	0.2738

Figure S11. Western blots (full-uncropped) and reading density number of the band for the protein of interest (p21 and PUMA) detected from Mortaparib^{Plus}-treated and control T47D cell lysates (Figure 2C). β-actin was used as an internal loading control.

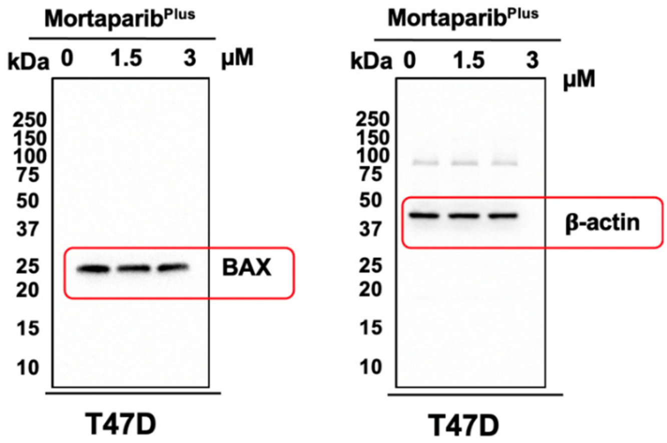

BAX (N-20)	Average of reading density number (Normalized against β-actin band	SD	Fold change	p-value
0 µM	0.95385582	0.08389279	1.00000002	
1.5 µM	0.84540548	0.07635205	0.88630323	0.1731
3 µM	0.84008582	0.05725664	0.88072623	0.1244

Figure S12. Western blots (full-uncropped) and reading density number of the band for the protein of interest (BAX) detected from Mortaparib^{Plus}-treated and control T47D cell lysates (Figure 2C). β-actin was used as an internal loading control.

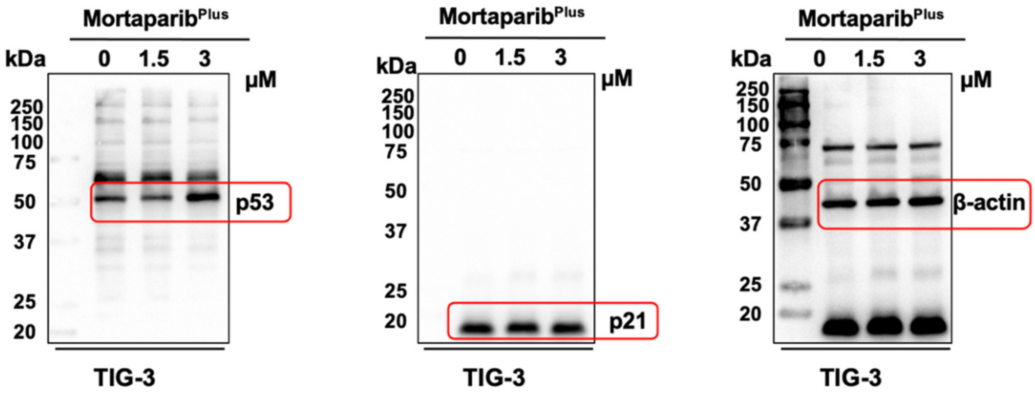

p53	Average of reading density number (Normalized against β-actin band)	SD	Fold change	p-value
0 µM	0.7023157	0.09191235	0.99999957	
1.5 µM	0.70731756	0.05166794	1.00712153	0.9526
3 µM	0.92516155	0.06669517	1.31730098	0.109

p21	Average of reading density number (Normalized against β-actin band)	SD	Fold change	p-value
0 µM	0.77692358	0.03279513	1.00000075	
1.5 µM	0.81999376	0.06959226	1.05543762	0.5115
3 µM	0.77260649	0.06433423	0.9944441	0.9403

Figure S13. Western blots (full-uncropped) and reading density number of the band for the protein of interest (p53 and p21) detected from Mortaparib^{Plus}-treated and control TIG-3 cell lysates (Figure 2D). β-actin was used as an internal loading control.

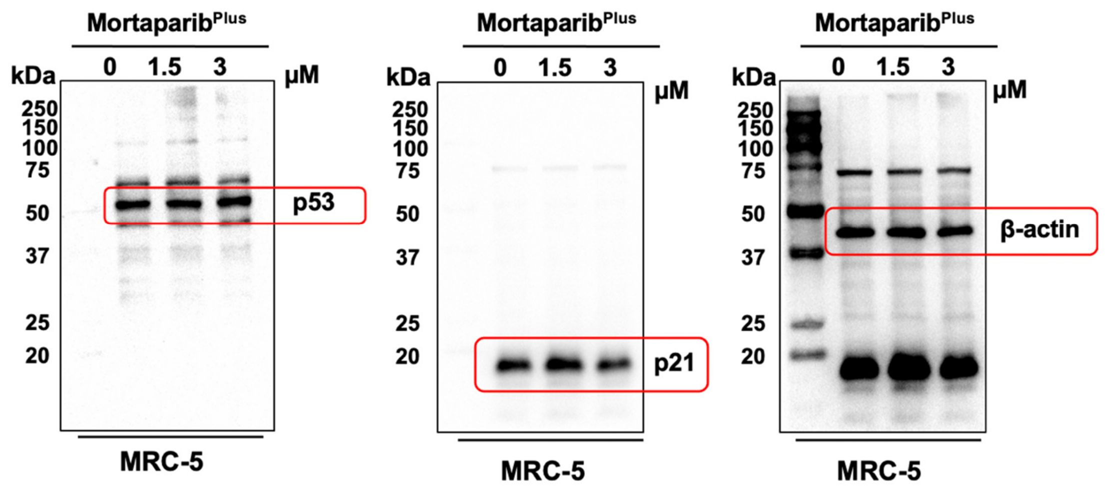

p53	Average of reading density number (Normalized against β-actin band)	SD	Fold change	p-value
0 μM	0.81750992	0.06659857	1.00000002	
1.5 μM	0.8423516	0.06215647	1.03038703	0.7369
3 μM	1.0202754	0.08122237	1.24802819	0.1121

p21	Average of reading density number (Normalized against β-actin band)	SD	Fold change	p-value
0 μM	0.98326805	0.07070277	0.99999995	
1.5 μM	1.06185643	0.0573642	1.07992564	0.3466
3 μM	0.92057238	0.05739709	0.93623741	0.4329

Figure S14. Western blots (full-uncropped) and reading density number of the band for the protein of interest (p53 and p21) detected from Mortaparib^{Plus}-treated and control MRC-5 cell lysates (Figure 2D). β-actin was used as an internal loading control.

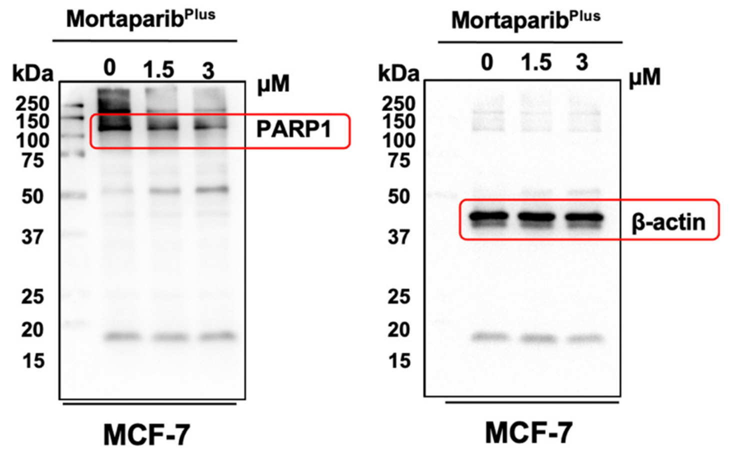

PARP1	Average of reading density number (Normalized against β-actin band	SD	Fold change	p value
0 μM	0.88438511	0.0334889	1.000005783	
1.5 μM	0.68692232	0.03966054	0.776727557	0.0329
3 μM	0.60210875	0.00094051	0.680825837	0.007

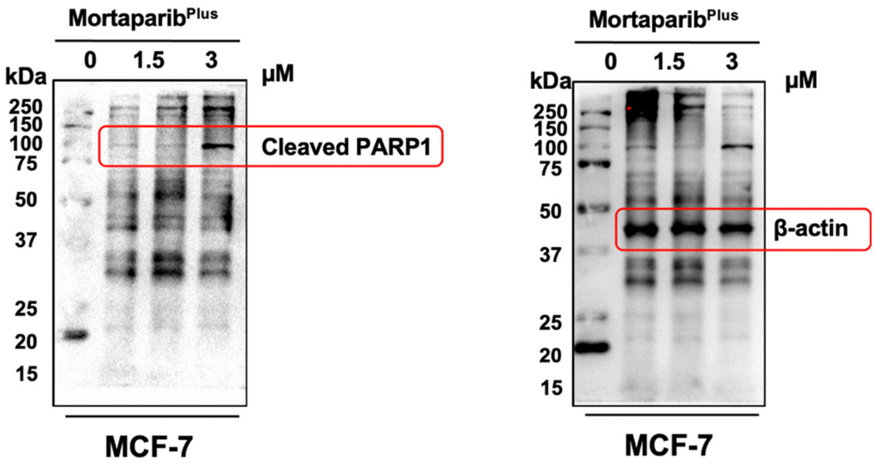

Cleaved PARP1	Average of reading density number (Normalized against β-actin band	SD	Fold change	p-value
0 μM	0.49753738	0.03402302	0.995074767	
1.5 μM	0.4649925	0.07401856	0.929985005	0.535
3 μM	0.76051633	0.07528891	1.521032653	0.05

Figure S15. Western blots (full-uncropped) and reading density number of the band for PARP1 and cleaved PARP1 detected from Mortaparib^{Plus}-treated and control MCF-7 cell lysates (Figure 5A). β-actin was used as an internal loading control.

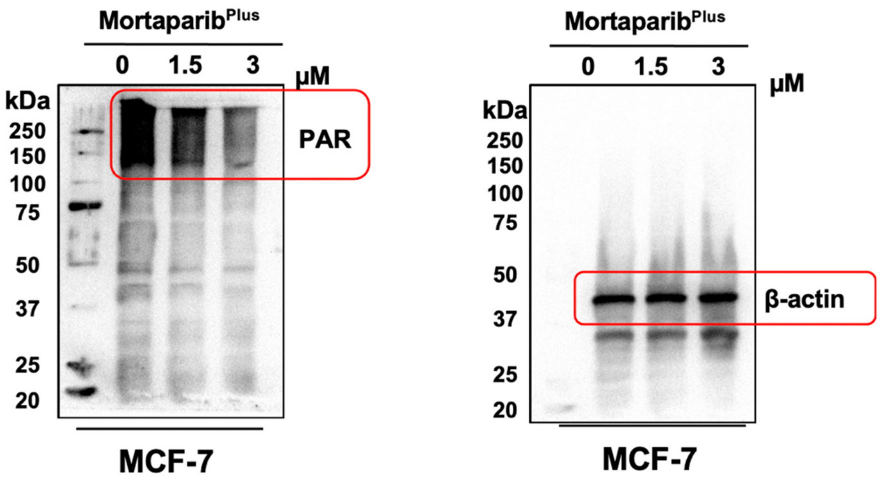

PAR	Average of reading density number (Normalized against β-actin band	SD	Fold change	p-value
0 µM	1.07	0.09	1.05	
1.5 µM	0.90	0.05	0.88	0.1446
3 µM	0.61	0.09	0.60	0.0362

Figure S16. Western blots (full-uncropped) and reading density number of the band for PAR detected from Mortaparib^{Plus}-treated and control MCF-7 cell lysates (Figure 5A). β-actin was used as an internal loading control.

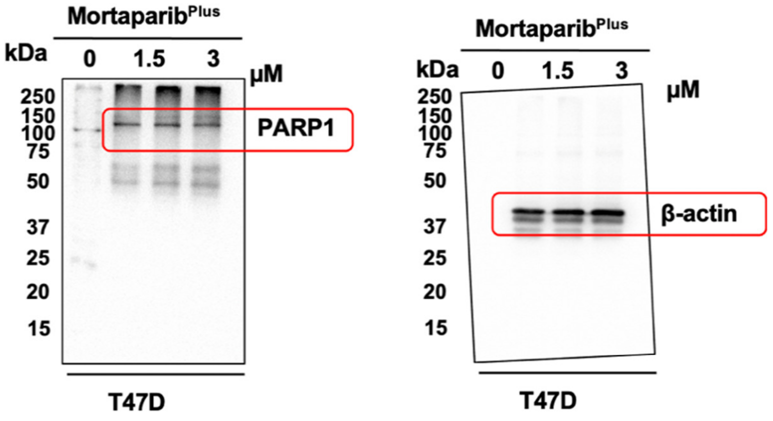

PARP1	Average of reading density number (Normalized against β-actin band)	SD	Fold change	p-value
0 μM	1.01794709	0.08461303	1.00000696	
1.5 μM	1.07801292	0.12035154	1.05901421	0.4454
3 μM	0.96903112	0.08066272	0.95195308	0.4347

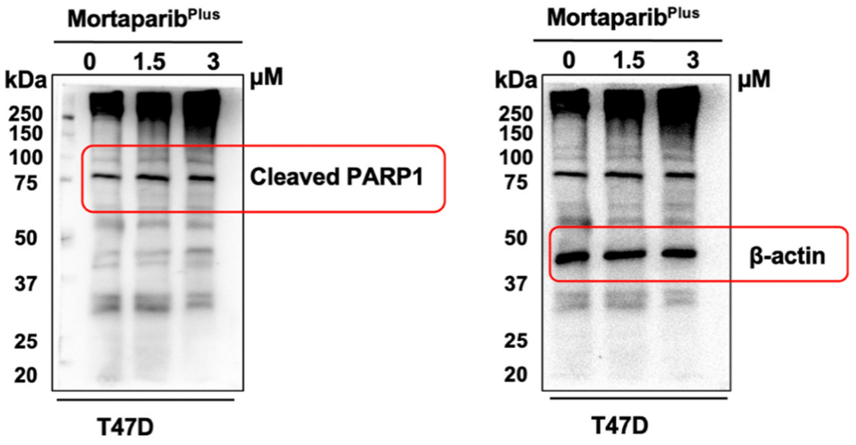

Cleaved PARP1	Average of reading density number (Normalized against β-actin band	SD	Fold change	p-value
0 μM	1.04946643	0.03237194	0.9999966	
1.5 μM	1.0819286	0.1275338	1.03092856	0.7605
3 μM	1.53694102	0.15343426	1.46449257	0.048

Figure S17. Western blots (full-uncropped) and reading density number of the band for PARP1 and cleaved PARP1 detected from MortaparibPlus-treated and control T47D cell lysates (Figure 6A). β-actin was used as an internal loading control.

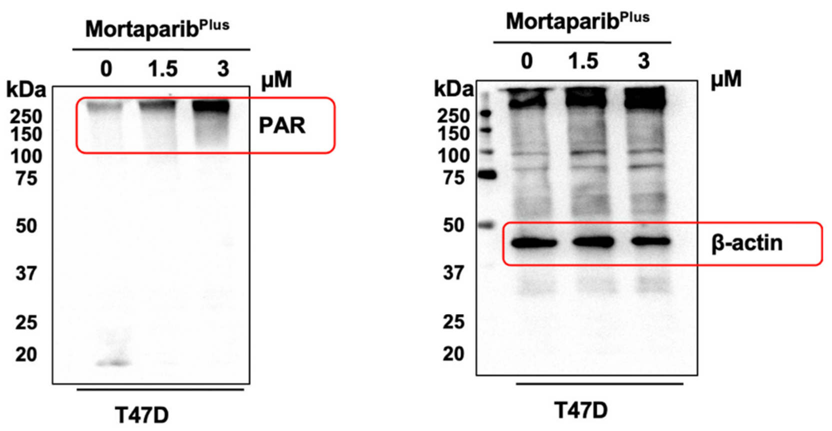

PAR	Average of reading density number (Normalized against β-actin band)	SD	Fold change	p-value
Control	0.25117421	0.03365634	1.00001675	
1.5 μM	0.48524151	0.03001412	1.93192462	0.0181
3 μM	1.22730696	0.00988831	4.88635969	0.0006

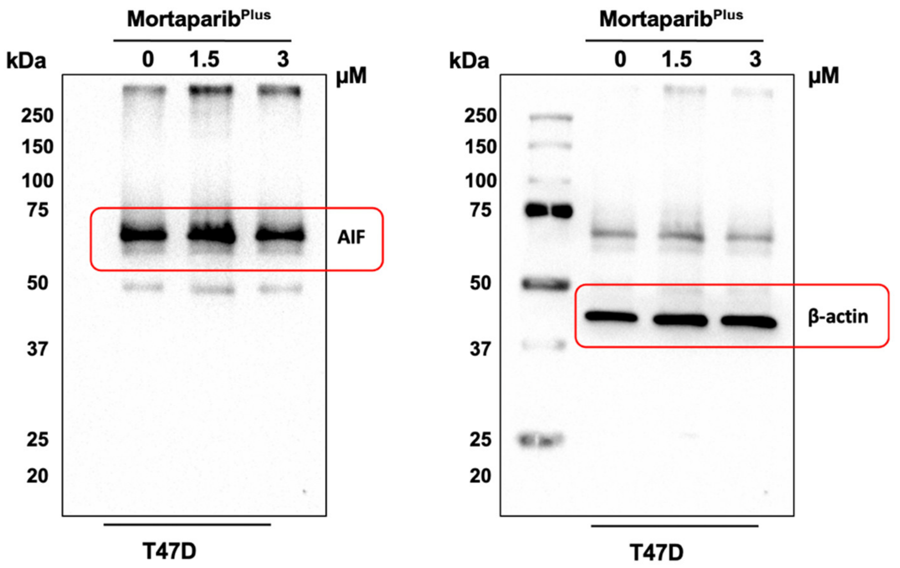

AIF	Average of reading density number (Normalized against β-actin band)	SD	Fold change	p-value
0 μM	1.39300775	0.20233626	1.00209175	
1.5 μM	1.51841604	0.11060577	1.09230706	0.5222
3 μM	1.22995181	0.01472693	0.88479376	0.3735

Figure S18. Western blots (full-uncropped) and reading density number of the band for PAR (Figure 6A) and AIF (Figure 7A) detected from Mortaparib^{Plus}-treated and control T47D cell lysates. β-actin was used as an internal loading control.

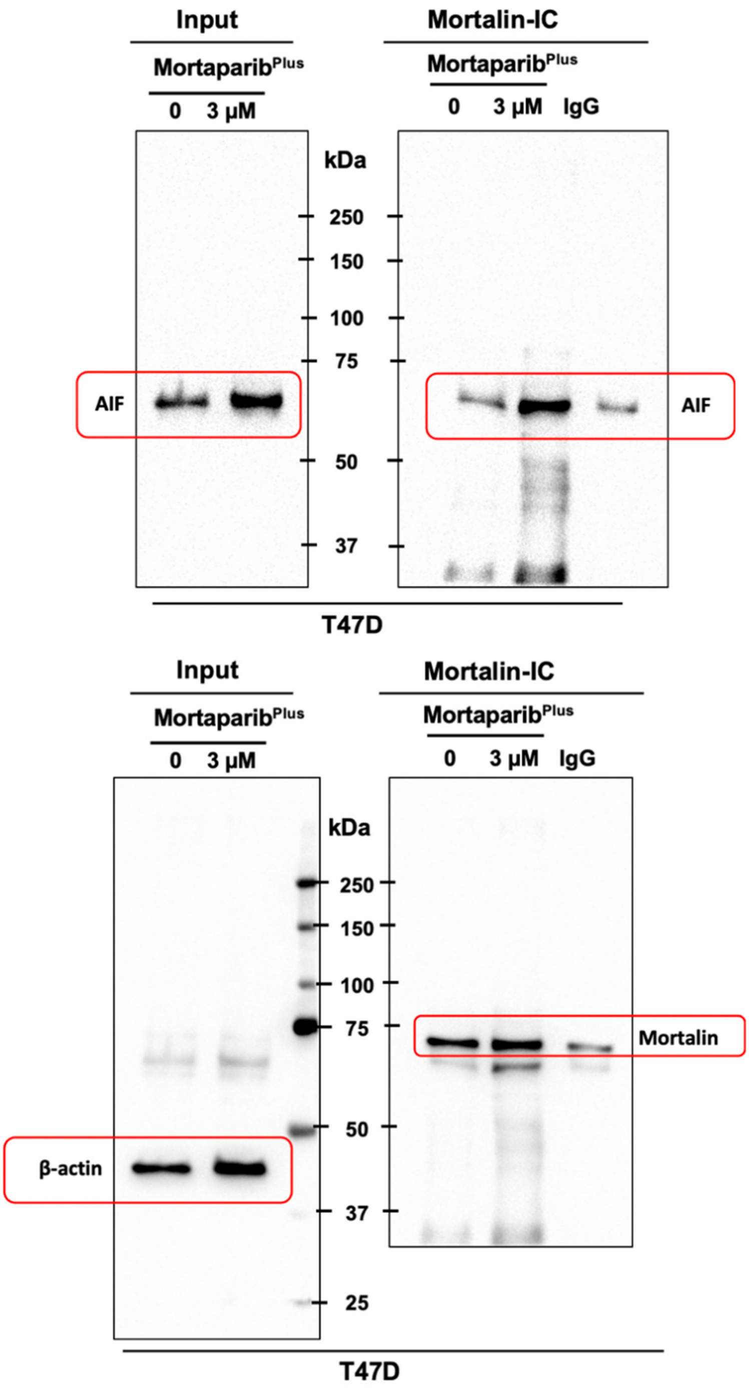

AIF	Average of reading density number (Normalized against mortalin band	fold	SD	p-value
0 μM	0.39395994	0.0312719	1.00015217	
3 μM	0.86068148	0.03784389	2.18502533	0.0055

Figure S19. Western blots (full-uncropped) and reading density number of the band for the protein of interest (AIF) detected from both mortalin immunocomplexes and inputs of Mortaparib^{Plus}-treated and control T47D cell lysates (Figure 8A). For the immunoprecipitated samples, mortalin bands were used to normalize an equal immunocomplexes. For the input samples, β-actin was used as an internal loading control.
